# The Diversity of the Genus *Tuber* in Greece—A New Species to Science in the Maculatum Clade and Seven First National Records

**DOI:** 10.3390/jof11050358

**Published:** 2025-05-05

**Authors:** Vassileios Daskalopoulos, Elias Polemis, Georgios Konstantinidis, Vasileios Kaounas, Nikolaos Tsilis, Vassiliki Fryssouli, Vassili N. Kouvelis, Georgios I. Zervakis

**Affiliations:** 1 Laboratory of General and Agricultural Microbiology, Department of Crop Science, Agricultural University of Athens, Iera Odos 75, 11855 Athens, Greece; vassilismks@gmail.com (V.D.); eliasp@ath.forthnet.gr (E.P.); vfrisouli@gmail.com (V.F.); 2 Greek Mushroom Society, Agiou Kosma 25, 51100 Grevena, Greece; manitarock@hotmail.gr (G.K.); bkaounas@gmail.com (V.K.); nicktsilis@gmail.com (N.T.); 3 Department of Biology, Section of Genetics and Biotechnology, National and Kapodistrian University of Athens, Panepistemiopolis, 15771 Athens, Greece; kouvelis@biol.uoa.gr

**Keywords:** Ascomycota, ectomycorrhizal fungi, ascomycete phylogeny, mushroom diversity, truffle, new species to science

## Abstract

Ectomycorrhizal fungi of the genus *Tuber* (Ascomycota) produce hypogeous ascomata commonly known as truffles. Despite their high ecological and economic importance, a considerable gap of knowledge exists concerning the diversity of *Tuber* species in the eastern Mediterranean region. In the frame of this study, more than 200 *Tuber* collections, originating from various regions of Greece, were examined. A new species to science, i.e., *Tuber leptodermum*, is formally described. *Tuber leptodermum* is grouped in the Maculatum clade, as revealed by the ITS and LSU rDNA concatenated phylogenetic tree, and appears as sister to *T. foetidum*. In addition, *T. leptodermum* exhibits distinct morphoanatomic features: it produces medium-sized, dark-brown ascomata with a thin pseudoparenchymatous peridium, composed of globose-to-angular cells and forms one-to-four-spored asci containing reticulate–alveolate, ellipsoid ascospores with broad meshes. Thirty other phylogenetic species are identified: seven of them (i.e., *T. anniae*, *T. buendiae*, *T. conchae*, *T. dryophilum*, *T. monosporum*, *T. regianum* and *T. zambonelliae*) constitute new records for the Greek mycobiota, while the presence of five other species is molecularly confirmed for the first time. Moreover, the existence of ten undescribed phylogenetic species is revealed, six of which are reported for the first time in Greece. Several taxonomic and phylogenetic issues and discrepancies in the genus *Tuber* are discussed in relation to the new findings.

## 1. Introduction

The term “true truffles” is used to describe the subterranean spherical and enclosed ascomata produced by fungi of the genus *Tuber* P.Micheli ex F.H.Wigg. (Pezizales, Ascomycota), which includes several gastronomically valuable and highly prized species [1,2,3]. These fungi form obligate ectomycorrhizal (ECM) relationships with various plant genera, which link the presence of truffles to that of their plant symbionts, depending on species-specific and environmental factors [4]. In addition, since truffle spores are mainly dispersed through the consumption of ascocarps by mycophagous animals, their distribution range is spatially limited [1,5]. During the last glacial periods, survival refugia for various species were established in favorable areas of southern Europe (e.g., in the Balkan peninsula); these refugia became the cradle for species recolonization of the continent [6]. Truffles follow a similar pattern of distribution, which is also affected by their unique biology and past biogeographic incidents [4,7,8,9].

The genus *Tuber* includes approx. 200 species which are grouped into 13 major phylogenetic clades, i.e., Aestivum, Excavatum, Gennadii, Gibbosum, Latisporum, Macrosporum, Maculatum, Melanosporum, Multimaculatum, Puberulum, Regianum, Rufum and Tumericum-Japonicum [1,10,11]. Several studies have dealt with the diversity of *Tuber* species in Europe; however, some regions—like the southern Balkans—still remain relatively understudied [2,12,13,14].

Regarding Greece in particular, the earliest documented reference of a “truffle” species can be found in the monumental work *Flora Graeca* [15], where “*Tuber album*” is mentioned; this finding, however, corresponds to *Terfezia arenaria* (Moris) Trappe, according to Chatin [16]. Xavier Laderer [17], a pharmacist at the palace during the reign of King Otto, reported the existence of *Tuber cibarium*. In addition, Chatin [16] described *Terfezia gennadii* based on collections made by Panagiotis Gennadios in the Peloponnese; this species was subsequently transferred to the genus *Tuber*, as *T. gennadii* (Chatin) Pat. Later, the first record of *Tuber aestivum* Vittad. was included in the *Fungus-host index for Greece* [18].

Hence, until the end of the 20th century, the diversity of the genus *Tuber* in Greece remained largely unknown, as reflected in the first national checklist of larger Ascomycota [19], which included the aforementioned records only. However, in recent years, the collection of truffles has become popular, while pioneering members of citizen science associations (e.g., the Greek Mushroom Society, GMS) have started identifying specimens using traditional taxonomy. A significant change in the knowledge of the genus *Tuber* in Greece is apparent from 2007 onwards, as reflected by the publication of a species checklist [20] and mushroom field guides [21,22,23,24,25]. The first study using DNA sequencing dealt with truffle specimens identified as *T. borchii* Vittad., *T. gennadii*, *T. oligospermum* (Tul. & C. Tul.) Trappe and *T. puberulum* Berk. & Broome [26], and was followed by reports of the presence of *T. brumale* Vittad. haplogroup II [8], *T. magnatum* Picco [9], *T. mesentericum* Vittad., *T. bituminatum* Berk. & Broome, *T. suave* Pacioni & M. Leonardi [27] and *T. magentipunctatum* Z. Merényi, I. Nagy, Stielow & Bratek [28]. In addition to these findings, two new species to science found in Greece have recently described, i.e., *T. pulchrosporum* Konstantinidis, Tsampazis, Slavova, Nakkas, Polemis, Fryssouli & Zervakis from material collected in spring to early summer in Bulgaria and continental Greece [29], and *T. aereum* Polemis, Daskalopoulos and Zervakis from specimens found in autumn and winter in Naxos Island [30]. At present, 26 species (including 4 phylogenetic species yet to be formally described) of this genus are known to exist in Greece, while the presence of only 15 of them is supported by molecular approaches.

The objective of the present work was to assess the diversity and distribution of the genus *Tuber* in Greece by examining numerous collections from a large range of geographic areas and a variety of habitats, through the combined use of morphoanatomical characters, ecological features and phylogenetics.

## 2. Materials and Methods

### 2.1. Specimens Studied

The biological material examined in the frame of this study was derived from the following sources: (a) from dried specimens previously deposited in the fungarium of the Laboratory of General and Agricultural Microbiology, Agricultural University of Athens (ACAM), or in personal collections of the GMS’s members [G. Konstantinidis (GK), V. Kaounas (VK), N. Tsilis (NT) and M. Gkilas (MG)]; and (b) from fresh ascomata sampled either by the authors or by collaborating truffle collectors. All the fresh samples received were subsequently dried and stored in the fungarium ACAM. In total, 242 collections (several of them including more than one specimen) were identified through the combined use of morphoanatomic criteria and DNA sequencing, while 63 of them were further subjected to phylogenetic analysis (Table 1; Supplementary Material, Table S1).

### 2.2. Morphoanatomic Features

Each sample, fresh or dried, was macroscopically examined and photographed using a Nikon SMZ18 stereoscope (Nikon Corporation, Tokyo, Japan) with the OCULAR vers. 2.0 software (Teledyne Photometrics, Tucson, AZ, USA). Color description was based on the “Flora of British Fungi: Colour Identification Chart” [31], and the respective codes are provided in braces. Microscopic observations were performed in water and in 3–5% (*w*/*v*) potassium hydroxide (KOH). Microanatomical features of the ascomata were examined using an Olympus BX53F2 (Olympus Corporation, Tokyo, Japan) or a Zeiss AxioImager A2 (Carl Zeiss Microscopy, Oberkochen, Germany) microscope, and were photographed with either an Olympus DP74 camera using the cellSens Entry software (Olympus Life Science, Waltham, MA, USA), or with an Axiocam 305 color camera using the ZEN vers. 2.3 lite software (Carl Zeiss Microscopy, Oberkochen, Germany), respectively. Photographs were taken under bright field, phase contrast and/or differential interference contrast (DIC) microscopy.

Initial evaluation of *Tuber* collections was based on their morphoanatomic and ecological features, in accordance with taxonomic keys included in various publications [27,28,32], and with a monograph on European *Tuber* species [12]. For selected specimens (e.g., those representing new species to science, and those corresponding to the first national records) the following microscopic features were thoroughly examined: the peridium width (total, and external if present), asci and stalk size, size of parenchymatic cells (if present), ascospore number per ascus, ascospore dimensions (per ascus category and in total), height of meshes or spines, and (in reticulate ascospores) number of meshes across the ascospore length. As a general rule, a minimum of 30 elements were measured for each feature by using Piximètre vers. 5.10 software (http://www.piximetre.fr/; France; accessed on 1 February 2025). Dimensions of various features are provided as follows: (minimum), range of length, (maximum), while the quotient (Q) value for ascospores is also provided, i.e., length divided by width. Average values (Av) were also calculated in the case of asci and ascospores; in addition, percentages are provided for the number of ascospores per ascus, and ranges are provided for the number of meshes along the spore length. Especially in the case of *T. regianum* Montecchi & Lazzari, the ascospore volume is also provided in accordance with Merenyi et al. [33] and Cseh et al. [28]: 0.523 × (width)^2^ × length.

### 2.3. DNA Extraction, Amplification and Sequencing of Phylogenetically Informative Markers

The total genomic DNA was isolated from dried or fresh material as follows: samples were ground to fine powder in the presence of liquid nitrogen, mixed with warm (60 °C) DNA lysis buffer (1% CTAB, 10 mM Na_2_EDTA, 0.7 M NaCl, and 50 mM Tris-HCl pH 8), and then extracted with phenol:chloroform:isoamyl alcohol (25:24:1), and precipitated with ethanol. The two regions of the nuclear ribosomal repeat unit, i.e., the internal transcribed spacer (ITS) and a fragment of the ribosomal large subunit gene (LSU), were amplified by the polymerase chain reaction (PCR) in a MiniAmp Plus Thermal Cycler (Applied Biosystems, CA, USA), using 0.8 μL of each primer (0.32 μM), 12.5 μL of FastGene^®^ Optima Polymerase hotstart ready mix (NIPPON Genetics Europe, Düren, Germany), 11.5 μL of UltraPure™ DNase/RNase-Free Distilled Water (Thermo Fisher Scientific, Waltham, MA, USA) and 1 μL of DNA template (ca. 10 ng μL^−1^). The ITS was amplified through the use of ITS1 and the ITS1f/ITS4 primer pair [34,35] under the following conditions: initial denaturation at 95 °C for 4 min, followed by 35 cycles at 95 °C for 30 sec, annealing at 55 °C for 30 sec, extension at 72 °C for 1 min and final extension at 72 °C for 7 min. In addition, partial sequences of the LSU were amplified by the primer pair LROR/LR5 [36] under the following conditions: initial denaturation at 95 °C for 4 min, followed by 35 cycles at 94 °C for 1 min, annealing at 52 °C for 50 sec, extension at 72 °C for 1 min and final extension at 72 °C for 10 min. Electrophoresis was performed with 1% (*w*/*v*) agarose gel and purification of the PCR product was performed with the PureLink Quick PCR kit (Invitrogen, Thermo Fisher Scientific, Waltham, MA, USA), and the purified products were quantified using the MULTISKAN SkyHigh Microplate Spectrophotometer (Thermo Fisher Scientific, Waltham, MA, USA). Sequences were obtained by using the same forward and reverse primers (as before in the amplification procedure) in an automated ABI sequencer (Life Technologies, USA) at CeMIA SA (Greece). FinchTV vers. 1.4.0 software (Geospiza, Inc., Denver, CO, USA) was used for chromatogram visualization and inspection of sequencing quality. Consensus sequences were generated using the SeqMan module of Lasergene vers. 7.1 software (DNAStar, Madison, WI, USA), manually edited to remove or replace all ambiguous characters, and cross-checked against public databases, including the international nucleotide sequence database collaboration (INSDC) [37] and UNITE [38]. Validated sequences were submitted to GenBank, and the pertinent accession numbers appear in Table 1 and in Supplementary Table S1.

**Table 1:** Details of biological material from the genus *Tuber* used in the phylogenetic analyses performed in the frame of this study: the phylogenetic clade and taxon, collection code, geographic origin, GenBank accession no. for ITS and LSU sequences, and respective references. The material from which sequences were generated for the first time is presented in **bold** typeface, while material corresponding to type specimens is underlined.

	CLADE/Taxon	Collection Code	Origin	ITS	LSU	Reference
	AESTIVUM					
1	*T. aestivum*	AQUI 10150	Italy	MZ423173		[32]
2	*T. aestivum*	S8	Slovakia	HQ285310		[39]
3	*T. aestivum*	2PL-M(u)	Poland	KX028767		[40]
4	*T. aestivum*	ACAMTub361	Greece	PP725740		[41]
5	***T. aestivum***	**ACAMTub271**	**Greece**	**PP918676**		**This study**
6	***T. aestivum***	**ACAMTub376**	**Greece**	**PP918694**		**This study**
7	*T. bituminatum*	AQUI 9833	Italy	OL711611		[27]
8	*T. bituminatum*	AQUI 8579	Greece	OL711613		[27]
9	***T. bituminatum***	**ACAMTub310**	**Greece**	**PP918764**		**This study**
10	***T. bituminatum***	**ACAMTub421**	**Greece**	**PP918781**		**This study**
11	*T. magnatum*	TO HG3458	Italy	MZ423175		[32]
12	*T. magnatum*	HHD19491	Turkey	PP239641		Unpublished
13	*T. magnatum*	B1	Italy	AJ586268		[42]
14	***T. magnatum***	**ACAMTub055**	**Greece**	**PP918868**		**This study**
15	***T. magnatum***	**ACAMTub156**	**Greece**	**PP918870**		**This study**
16	*T. mesentericum*	AQUI 9717	Italy	OL711593		[27]
17	*T. mesentericum*	AQUI 10238	France	OL711600		[27]
18	*T. mesentericum*	AQUI 10230	Greece	OL711607		[27]
19	***T. mesentericum***	**ACAMTub111**	**Greece**	**PP918880**		**This study**
20	***T. mesentericum***	**ACAMTub250**	**Greece**	**PP918885**		**This study**
21	*T. panniferum*	JT12835	Spain	HM485380		[43]
22	*T. panniferum*	MUB:Fung-0973	Spain	MN962725		[44]
23	***T. panniferum***	**ACAMTub269**	**Greece**	**PP918896**		**This study**
24	***T. panniferum***	**ACAMTub385**	**Greece**	**PP918897**		**This study**
25	*T. pulchrosporum*	VN091	Greece	MK113975		[29]
26	*T. pulchrosporum*	1961 F0388	Bulgaria	MK113982		[29]
27	*T. suave*	AQUI 7131	Italy	OL711623		[27]
28	*T. suave*	AQUI 10229	Greece	OL711627		[27]
	EXCAVATUM					
29	*T. excavatum*	AP-T63a	Slovenia	FM205555		Unpublished
30	*T. excavatum*	Tub10	Italy	HM152012		Unpublished
31	***T.* aff. *excavatum* 1**	**ACAMTub151**	**Greece**	**PP918825**		**This study**
32	***T.* aff. *excavatum* 1**	**ACAMTub162**	**Greece**	**PP918826**		**This study**
33	*T. excavatum*	SFI:TUBEXC/251008B	Slovenia	FN433141		Unpublished
34	*T. fulgens*	AP-T60	Slovenia	FM205568		Unpublished
35	*T. excavatum*	zb3377	Hungary	HM152023		Unpublished
36	*T. excavatum*	AP-T11	Slovenia	FM205554		Unpublished
37	***T.* aff. *excavatum* 2**	**ACAMTub028**	**Greece**	**PP918828**		**This study**
38	***T.* aff. *excavatum* 2**	**ACAMTub316**	**Greece**	**PP918829**		**This study**
39	*T. excavatum*	SFI:TUBEXC/121008	Slovenia	FN433142		Unpublished
40	*T. excavatum*	SA1TE	Poland	KJ524533		Unpublished
41	***T.* aff. *excavatum* 3**	**ACAMTub168**	**Greece**	**PP918841**		**This study**
42	***T.* aff. *excavatum* 3**	**ACAMTub320a**	**Greece**	**PP918851**		**This study**
43	*T. excavatum*	BM100	Spain	FJ748899		[45]
44	*T. excavatum*	MA:Fungi:54695	Spain	FM205561		Unpublished
45	*T. fulgens*	zb3335_205	Hungary	HM152021		Unpublished
46	*T. fulgens*	CMI-Unibo_5034	Iran	MW884551		[46]
47	*T. fulgens*	HMT44	Italy	HM151979		Unpublished
48	***T. fulgens***	**ACAMTub170**	**Greece**	**PP918853**		**This study**
49	***T. fulgens***	**ACAMTub312**	**Greece**	**PP918854**		**This study**
50	*T. iranicum*	CMI-Unibo 4939	Iran	MN854634		[47]
51	*T. iranicum*	CMI-Unibo 4965	Iran	MN854635		[47]
	GENNADII					
52	*T. gennadii*	B M1904	Italy	HM485361		[43]
53	*T. gennadii*	AH39251	Spain	JN392211		[26]
54	***T.* aff. *gennadii***	**ACAMTub050**	**Greece**	**PP918857**		**This study**
55	***T.* aff. *gennadii***	**ACAMTub131**	**Greece**	**PP918858**		**This study**
56	*T. gennadii*	AH38957	Spain	JN392204		[26]
57	*T. gennadii*	VK2141	Greece	JN392205		[26]
58	*T. gennadii*	AH38955	Spain	JN392207		[26]
59	*T. gennadii*	MRG496	Spain	OQ565278		[48]
60	*T. conchae*	MRG486	Spain	OQ565276		[48]
61	***T. conchae***	**ACAMTub046**	**Greece**	**PP918816**		**This study**
62	*T. lacunosum*	AH38912	Morocco	JN392214		[26]
63	*T. lacunosum*	MRG692	Spain	OQ565280		[48]
64	*T. lucentum*	j957	Spain	MN437527		[49]
65	*T. lucentum*	MUB:Fung-j825	Spain	NR184909		[49]
	MACROSPORUM					
66	*T. calosporum*	HKAS:88790	China	KT444598		[50]
67	*T. calosporum*	HKAS:88751	China	KT444600		[50]
68	*T. glabrum*	BJTCFan228	China	KF002731		[51]
69	*T. glabrum*	BJTCFan232	China	KF002727		[51]
70	*T. macrosporum*	CMI-Unibo_4937	Iran	MW884553		[46]
71	*T. macrosporum*	CMI-Unibo_4938	Iran	MW884554		[46]
72	*T. macrosporum*	FHS-449	Serbia	FM205664		[14]
73	*T. macrosporum*	IC28092233	Spain	PP948901		Unpublished
74	*T. macrosporum*	ITA_014s	Italy	KP738351		[52]
75	***T. macrosporum***	**ACAMTub052**	**Greece**	**PP918859**		**This study**
76	***T. macrosporum***	**ACAMTub110**	**Greece**	**PP918860**		**This study**
77	***T. macrosporum***	**ACAMTub252**	**Greece**	**PP918862**		**This study**
78	*T. macrosporum*	ITA_003V	Italy	KP738380		[52]
79	*T. macrosporum*	ITA_008	Italy	KP738388		[52]
80	*T. macrosporum*	FRA_002	France	KP738361		[52]
81	***T. monosporum***	**ACAMTub455**	**Greece**	**PP918907**	**PP892729**	**This study**
82	*T. sinomonosporum*	BJTCFan150	China	KF002729		[51]
	MACULATUM					
83	*T. arnoldianum*	RH1619	USA	KU186913		[53]
84	*T. arnoldianum*	FLAS:F-71902	USA	OR762689	PP337424	Unpublished
85	*T. arnoldianum*	FLAS:F-71880	USA	OR762672	PP337406	Unpublished
86	*T. arnoldianum*	AMC105	USA	OP413000		Unpublished
87	*T. aztecorum*	ITCV 993 ITCV	Mexico	NR159044		[54]
88	*T. aztecorum*	GG993 FLAS	Mexico	KY271791		[54]
89	*T. beyerlei*	JT32597	USA	HM485408		[43]
90	*T. beyerlei*	Berch 0042	Canada	KP972075		[55]
91	*T. brennemanii*	MES653	USA	MF611779	KY565252	[56]
92	*T. brennemanii*	RH1746	USA	MF611791	KY565258	[56]
93	*T. excelsum-reticulatum*	BJTC FAN863	China	OM265272	OM366224	[11]
94	*T. excelsum-reticulatum*	BJTC FAN758	China	OM265264	OM366218	[11]
95	*T. foetidum*	ZB2489	Hungary	JQ288906	JF261362	Unpublished
96	*T. foetidum*	ZB2452	Hungary	JQ288905	JF261361	Unpublished
97	*T. foetidum*	JCAS0140001000096	Spain	MH703905		[57]
98	*T. foetidum*	ZB3454	Finland	FN568055		[58]
99	*T. foetidum*	B-2489	Hungary	AJ557544		[13]
100	*T. foetidum*	B-2452	Hungary	AJ557543		[13]
101	***T. leptodermum***	**ACAMTub475**	**Greece**	**PQ877422**	**PQ881954**	**This study**
102	***T. leptodermum***	**ACAMTub359**	**Greece**	**PP918908**	**PP905688**	**This study**
103	***T. leptodermum***	**ACAMTub379**	**Greece**	**PP918909**	**PP905689**	**This study**
104	***T. leptodermum***	**ACAMTub380**	**Greece**	**PP918910**	**PP905690**	**This study**
105	***T. leptodermum***	**ACAMTub382**	**Greece**	**PP918911**	**PP905691**	**This study**
106	*T. hubeiense*	HMAS 60233	China	KT067688	KT067694	[11]
107	*T. lusitanicum*	MUB:Fung-0986	Spain	MT621651	MT705332	[59]
108	*T. lusitanicum*	MUB:Fung-0988	Spain	MT621653		[59]
109	*T. lusitanicum*	MUB:Fung-0990	Spain	MT621655		[59]
110	*T. maculatum*		Italy	AF003919		Unpublished
111	*T. maculatum*	herbarium 1967	Italy	EU753269		Unpublished
112	*T. maculatum*	Imatra_3	Finland	MZ389968		[60]
113	*T. maculatum*	FLAB22	Uruguay	PP297668		[61]
114	*T. maculatum*	BJTC FAN876	China	OM265278	OM366228	[11]
115	*T. maculatum*	BJTC FAN868	China	OM265274	OM366227	[11]
116	*T. miquihuanense*	ITCV885	Mexico	HM485414	JF419292	[43]; [62]
117	*T. pseudomagnatum*	BJTC FAN163	China	JQ771192		[63]
118	*T. pseudomagnatum*	BJTC FAN299	China	OM265244	OM366195	[11]
119	*T. pseudomagnatum*	BJTC FAN392	China	KP276184	KP276193	[64]
120	*T. pseudomagnatum*	BJTC FAN390	China	OM265243		[11]
121	*T. pseudomagnatum*	BJTC FAN389	China	OM265242		[11]
122	*T. rapaeodorum*	BG Kew K(M)128884	England	EU784429		[65]
123	*T. rapaeodorum*	CMI-UNIBO 2483	Armenia	DQ011849		Unpublished
124	*T. walkeri*	RH5211	USA	JF419260		[62]
125	*T. walkeri*	FLAS:MES-274	USA	MT156441		Unpublished
126	*T. walkeri*	RH794	USA	JF419258	JF419308	[62]
127	*T. whetstonense*	JT25783	USA	HM485392	JF419304	[43]; [62]
128	*T. whetstonense*	SOC 756 OSC 111412	USA	AY830855		[66]
129	*T. whetstonense*	FLAS:MES-210	USA	MT156439		Unpublished
130	*T. wumengense*	BJTC FAN218A	China	KT067682	KT067707	[64]
131	*T. wumengense*	HMAS 60229	China	KT067687		[64]
132	*T. wumengense*	BJTC FAN586	China	OM265254		[11]
	MELANOSPORUM					
133	*T. brumale*	G1126	Greece	KF550998		[8]
134	*T. brumale*	BTb2p2	Turkey	KF551063		[8]
135	*T. brumale*	FHS-460	Serbia	FM205660		[14]
136	*T. brumale*	CMI-Unibo_5001	Iran	MW829419		[46]
137	*T. brumale*	MA:Fungi:28373	Spain	FM205550		[14]
138	*T. brumale*	TB59k1	France	KF550974		[8]
139	***T. brumale***	**ACAMTub159**	**Greece**	**OP850807**		**This study**
140	***T. brumale***	**ACAMTub233**	**Greece**	**PP918804**		**This study**
141	***T. brumale***	**ACAMTub456**	**Greece**	**PP918814**		**This study**
142	*T. cryptobrumale*	HNHM<HUN>:BP107922	Hungary	KU203777		[33]
143	*T. cryptobrumale*	HNHM<HUN>:BP107923	Hungary	KU203778		[33]
144	*T. indicum*	Tind-gs01	China	DQ375490		[67]
145	*T. indicum*	Tind-gs05	China	DQ375491		[67]
146	*T. melanosporum*	AQUI 10152	Italy	MZ423176		[32]
147	*T. melanosporum*	MEL178	France	EU555385		[68]
148	***T. melanosporum***	**ACAMTub256**	**Greece**	**PP918878**		**This study**
149	*T. petrophilum*	LJU-GIS-TUBsp_xx1010A	Serbia	HG810883		[69]
150	*T. petrophilum*	LJU-GIS-TUBsp_xx1010D	Serbia	HG810886		[69]
151	*T. thracicum*	SOMF:30882	Bulgaria	OR666020		[70]
152	*T. thracicum*	SOMF:30883	Bulgaria	OR666027		[70]
	PUBERULUM					
153	*T. oligospermum*	CMI-UNIBO 4230	Morocco	KF021624		[71]
154	*T. oligospermum*	CMI-UNIBO 4234	Morocco	KF021622		[71]
155	*T. oligospermum*	AH39002	Italy	JN392248		[26]
156	*T. oligospermum*	AH37861	Italy	JN392251		[26]
157	*T. oligospermum*	GK3060	Greece	JN392249		[26]
158	***T.* aff*. oligospermum* 1**	**ACAMTub075**	**Greece**	**PP918887**		**This study**
159	*T. oligospermum*	MUB:Fung-1049	Spain	PQ281432		Unpublished
160	*T. oligospermum*	MUB:Fung-1050	Israel	PQ281433		Unpublished
161	*T. oligospermum*	GK5388	Greece	JN392263		[26]
162	***T.* aff*. oligospermum* 2**	**ACAMTub062**	**Greece**	**PP918889**		**This study**
163	*T. oligospermum*	HAI-D-102	Israel	JN392271		[26]
164	*T. oligospermum*	VK2042	Greece	JN392254		[26]
165	Soil sample	S052	Iran	UDB0765470		Unpublished
166	***T.* aff*. oligospermum* 3**	**ACAMTub133**	**Greece**	**PP918893**		**This study**
167	*T. anniae*	JT13209	USA	HM485338		[43]
168	*T. anniae*	Juva_1	Finland	MZ389970		[60]
169	***T. anniae***	**ACAMTub024**	**Greece**	**PP918746**		**This study**
170	*T. borchii*	AP-T30	Slovenia	FM205497		[14]
171	*T. borchii*	CMI-Unibo_5047	Iran	MW829422		[46]
172	*T. borchii*	17Bo	Italy	DQ679802		[72]
173	***T. borchii***	**ACAMTub135**	**Greece**	**PP918787**		**This study**
174	***T. borchii***	**ACAMTub334**	**Greece**	**PP918792**		**This study**
175	***T. borchii***	**ACAMTub371A**	**Greece**	**PP918796**		**This study**
176	*T. borchii*	AQUI 10151	Italy	MZ423174		[32]
177	*T. borchii*	GK5597	Greece	JN392228		[26]
178	*T. borchii*	CMI-UNIBO 3026	Italy	FJ554493		[72]
179	*T. borchii*	MUB:Fung-1036	Spain	PQ273773		Unpublished
180	***T. borchii***	**ACAMTub120A**	**Greece**	**PP918786**		**This study**
181	***T. borchii***	**ACAMTub207**	**Greece**	**PP918802**		**This study**
182	***T. borchii***	**ACAMTub377**	**Greece**	**PP918803**		**This study**
183	*T. dryophilum*		Italy	AF003917		Unpublished
184	*T. puberulum*	GK4352	Greece	JN392223		[26]
185	*T. dryophilum*	GB69	Italy	HM485353		[10]
186	*T. puberulum*	GK5601	Greece	JN392224		[26]
187	***T. dryophillum***	**ACAMTub043**	**Greece**	**PP918819**		**This study**
188	***T. dryophillum***	**ACAMTub134**	**Greece**	**PP918821**		**This study**
189	***T. dryophillum***	**ACAMTub182**	**Greece**	**PP918822**		**This study**
190	***T. dryophillum***	**ACAMTub204**	**Greece**	**PP918824**		**This study**
191	*T. lijiangense*	BJTC FAN307	China	KP276188		[64]
192	*T.* cf. *lijiangense*	BJTC FAN148	China	OM286798		[11]
193	*T. puberulum*	Berk. & Broome TL11885	Denmark	AJ969626		[73]
194	*T. puberulum*	Berk. & Broome TL3857	Denmark	AJ969625		[73]
	REGIANUM					
195	*T. magentipunctatum*	ZB4293	Hungary	JQ288909		[74]
196	*T. magentipunctatum*	MO793	Italy	KY420089		[28]
197	*T. magentipunctatum*	GK 4552	Greece	KY420088		[28]
198	***T. magenctipunctatum***	**ACAMTub126**	**Greece**	**PP918865**		**This study**
199	***T. magenctipunctatum***	**ACAMTub381**	**Greece**	**PP918866**		**This study**
200	*T. regianum*	M46	Spain	KY420100		[28]
201	*T. regianum*	ZB3081	Slovakia	KY420098		[74]
202	*T. regianum*	IC13071106	Spain	KY420103		[74]
203	***T. regianum***	**ACAMTub226**	**Greece**	**PP918898**		**This study**
	RUFUM					
204	*T. aereum*	ACAMTub245	Greece	PP425915		[30]
205	***T. aereum***	**ACAMTub467**	**Greece**	**PQ336045**		**This study**
206	*T. nitidum*	AH39101	Morocco	JX402092		[75]
207	*T. nitidum*	MUB:Fung-0934	Spain	MN962721		[76]
208	*T. rufum*	SFI:TUBRUF/111208	N. Macedonia	FN433159		Unpublished
209	***T. nitidum***	**ACAMTub039**	**Greece**	**PP918707**		**This study**
210	***T. nitidum***	**ACAMTub375**	**Greece**	**PP918708**		**This study**
211	*T. rufum*	1798	Italy	EF362473		[77]
212	*T. rufum*	17114	Italy	JF908745		[78]
213	*T. rufum* f. *ferrugineum*	FHS-393	Serbia	FM205676		[14]
214	***T.* aff. *rufum* 1**	**ACAMTub251**	**Greece**	**PP918918**		**This study**
215	*T. rufum*	1785	Italy	EF362475		[77]
216	*T. rufum*	FHS-XX12	Serbia	FM205690		[14]
217	*Tuber* sp. 57	JT13224	USA	JQ925650		[10]
218	***T.* aff. *rufum* 2**	**ACAMTub315**	**Greece**	**PP918919**		**This study**
219	*T. buendiae*	MUB:Fung-0974	Spain	MT006095		[76]
220	***T. buendiae***	**ACAMTub350**	**Greece**	**PP918815**		**This study**
221	*T. melosporum*	AH31737	Spain	JX402095		[75]
222	*T. melosporum*	MA-33360	Spain	OQ866602		Unpublished
223	*T. pustulatum*	AQUI 9725	Spain	MK211278		[79]
224	*T. rufum*	MA:Fungi:28397	Spain	FM205615		[14]
225	*Tuber* sp.	TUF113700	Estonia	UDB027742		Unpublished
226	*T. rufum* f. *lucidum*	CMI-Unibo_4944	Iran	MW829417		[46]
227	*T. rufum* f. *lucidum*	AZ2097	Italy	FJ809888		[80]
228	*T. rufum*	GK4422	Greece	JX402094		[75]
229	***T.* aff. *rufum* 3**	**ACAMTub425**	**Greece**	**PP918727**		**This study**
230	***T.* aff. *rufum* 3**	**ACAMTub067**	**Greece**	**PP918709**		**This study**
231	***T.* aff. *rufum* 3**	**ACAMTub230**	**Greece**	**PP918710**		**This study**
232	***T.* aff. *rufum* 3**	**ACAMTub317**	**Greece**	**PP918714**		**This study**
233	*T. zambonelliae*	MUB:Fung-0995	Spain	MW632951		[81]
234	*T. zambonelliae*	MUB:Fung-1007	Spain	MW632955		[81]
235	***T. zambonelliae***	**ACAMTub040**	**Greece**	**PP918921**		**This study**
236	***T. zambonelliae***	**ACAMTub079**	**Greece**	**PP918922**		**This study**
	OUTGROUP					
237	*Choiromyces helanshanensis*	HKAS 80634	China	NR153899		[82]

ITS: internal transcribed spacer; LSU: large subunit 25-28S RNA.

### 2.4. Phylogenetic Analysis

For the phylogenetic analyses, sequences generated in this study, as well as reference sequences of *Tuber* species derived from the GenBank (https://www.ncbi.nlm.nih.gov/genbank/; accessed on 10 March 2025) and UNITE (https://unite.ut.ee/; accessed on 10 March 2025), were used (Table 1). Sequence alignments were implemented using the ClustalW algorithm of MEGA11 software [83]. To exclude poorly aligned or ambiguous regions of the sequences, trimming was performed manually. The phylogenetic trees were constructed with the maximum likelihood (ML) method via the IQ-TREE vers. 2.4.0 software by using the GTR + gamma substitution model, estimated as the best-fit model by the same software [84,85]. ML support values in the branches were obtained using ultrafast bootstrap (1000 replicates), implemented in the IQ-TREE software [86]. Bayesian inference (BI) analysis was performed utilizing the BEAST vers. 10.5.0 software [87], employing the same nucleotide substitution model as in the ML method. The Effective Sample Sizes (ESS > 200) were evaluated by the Tracer vers. 1.7.2 software [88]. A single chain of 10 million generations was run, logging every 100 generations. A maximum clade credibility (MCC) tree was generated using TreeAnnotator vers. 10.5.0 [87] with 10% burn-in. Visualization of the phylogenetic tree was performed with the iTOL vers. 6 web-based software [89]. Bootstrap support (≥70%) and BPP (≥0.95) values are shown in the tree nodes, while *Choiromyces helanshanensis* (sequence from the holotype) was used as an outgroup in all cases. The tree scale indicates the expected number of changes per site. The resulting phylogenetic trees are deposited in TreeBASE (http://www.treebase.org; accessed on 20 March 2025) with accession ID 32081.

Each taxonomic annotation appearing on the phylogenetic trees corresponds to a well-defined species or to a non-described phylospecies, which is provisionally named by using the epithet of the most related taxon and the term “aff.” (affinity). Although we acknowledge the limitations mentioned by earlier studies regarding the generalized use of the ITS region as a universal DNA barcode marker for fungi, we followed the widely adopted threshold for separating species of the genus *Tuber*, i.e., <96% for ITS sequence identity in pairwise comparisons [10,11,27]. The respective values were estimated with the aid of the BLASTn web-based tool [90], accessed through the NCBI website (https://blast.ncbi.nlm.nih.gov/Blast.cgi; accessed on 10 March 2025).

## 3. Results

### 3.1. Phylogeny of the Genus Tuber

**Figure 1:**
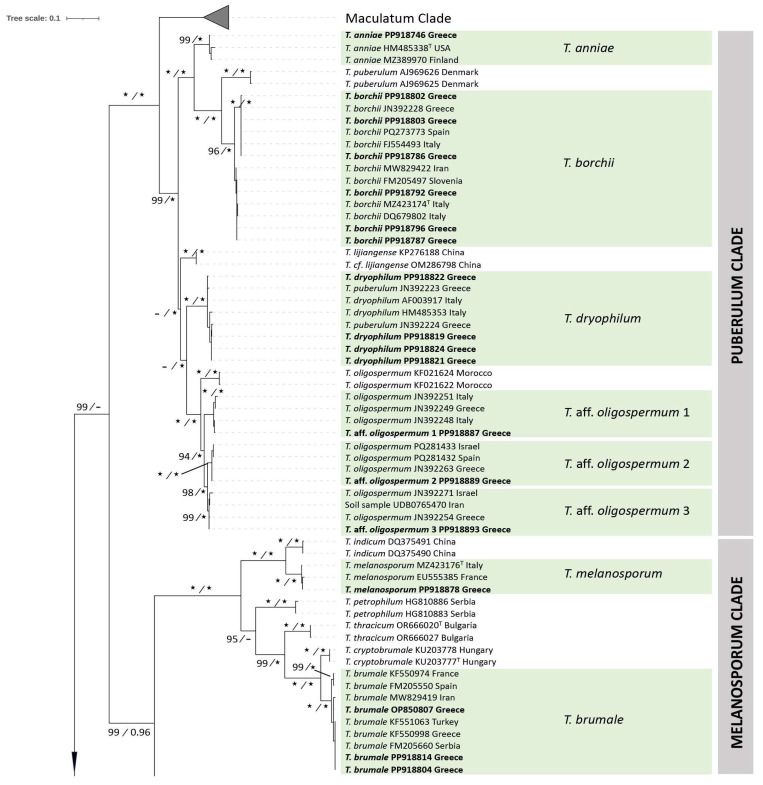

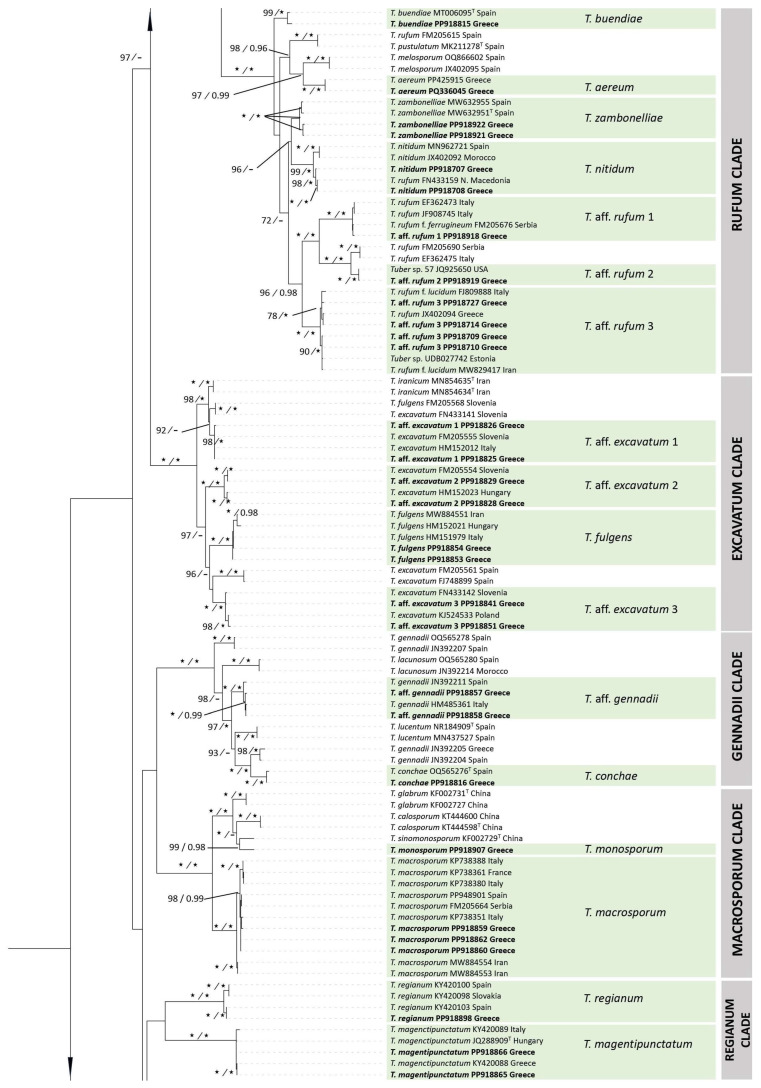

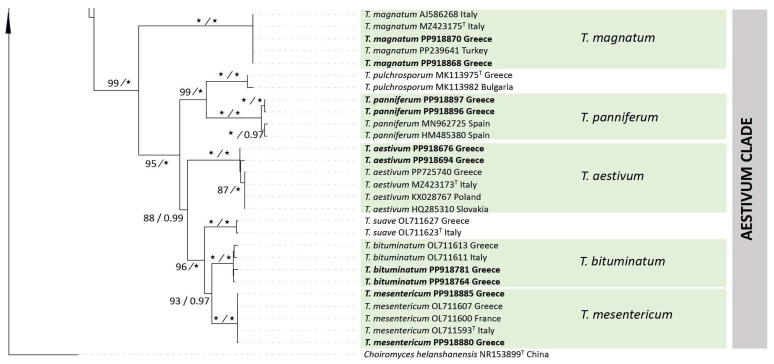
A maximum likelihood (ML) tree, based on ITS sequences, depicting the phylogeny of *Tuber* spp. in Greece. *Choiromyces helanshanensis* is used as an outgroup. Bootstrap support (>70%) for ML and Bayesian posterior probabilities (>0.95) are indicated at the tree nodes; values of 100% and 1.00, respectively, are marked by asterisks (*). Sequences generated in this study appear in bold typeface, while the Maculatum clade appears collapsed, and is presented in Figure 2, Figure 3 and Figure 4. Species including sequences generated by this study are marked in green. Clades are presented in the vertical line on the right side of the figure. The use of sequences from type specimens is indicated by “T” placed as superscript after the respective GenBank accession number.

**Figure 2:**
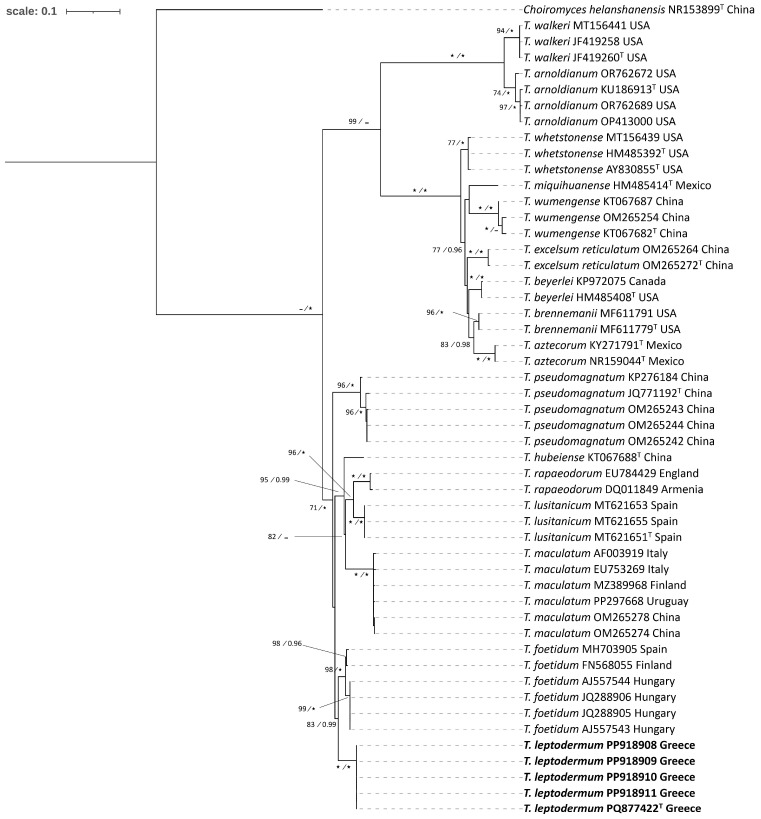
A maximum likelihood (ML) tree, based on ITS sequences, depicting the phylogeny of the Maculatum clade by including *T. leptodermum* sp. nov., which appears in bold typeface. *Choiromyces helanshanensis* is used as an outgroup. Bootstrap support (>70%) for ML and Bayesian posterior probabilities (>0.95) are indicated at the tree nodes; values of 100% and 1.00, respectively, are marked by asterisks (*). The use of sequences from type specimens is indicated by “T” placed as superscript after the respective GenBank accession number.

**Figure 3:**
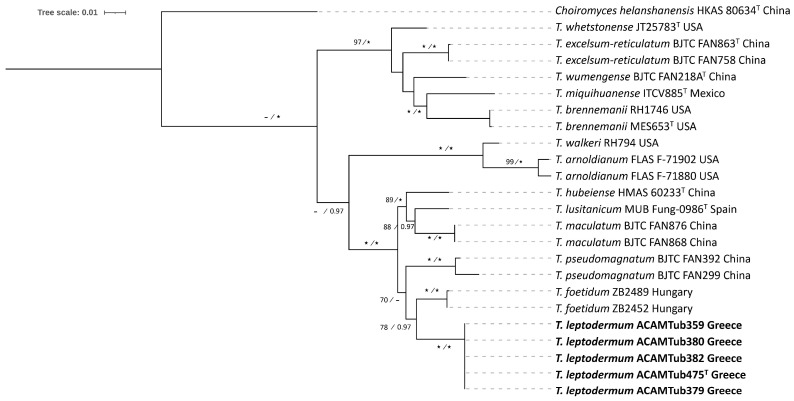
A maximum likelihood (ML) concatenated tree, based on ITS + LSU sequences, depicting the phylogeny of the Maculatum clade by including *T. leptodermum* sp. nov., which appears in bold typeface. *Choiromyces helanshanensis* is used as an outgroup. Bootstrap support (>70%) for ML and Bayesian posterior probabilities (>0.95) are indicated at the tree nodes; values of 100% and 1.00, respectively, are marked by asterisks (*). The use of sequences from type specimens is indicated by “T” placed as superscript after the respective GenBank accession number.

A total of 242 ITS and six LSU sequences were generated for the first time (Supplementary Material, Table S1); among them, 63 ITS and five LSU sequences were selected for inclusion in the phylogenetic trees of this study (Figure 1, Figure 2 and Figure 3, Supplementary Figure S1). In addition to the Maculatum clade, which appears compressed in the ML ITS tree (Figure 1), the following *Tuber* clades (as these were defined by Bonito et al. [10], and Bonito and Smith [1]) are included: Aestivum, Excavatum, Gennadii, Macrosporum, Melanosporum, Puberulum, Regianum and Rufum. They consist mostly of species of European origin and/or distribution, and contain 187 sequences (other than those of the Maculatum clade), 58 of which derive from this work and 129 of which are sequences deposited in NCBI and UNITE databases (16 of them correspond to specimens from Greece generated in the frame of previous studies) (Table 1). 

The length of the aligned ITS dataset was 566 nucleotides, and the resulting phylogenetic trees inferred from ML and BI analyses demonstrated consistent topologies (with no supported conflicts) showing high ML-BS and BPP support values.

In the Aestivum clade, specimens from this study are included in five highly supported groups (ML-BS 100%, BPP 1.00) which correspond to well-defined species, i.e., *T. aestivum*, *T. bituminatum*, *T. magnatum* Picco, *T. mesentericum* and *T. panniferum* Tul. & C. Tul. All of them, with the exception of *T. panniferum*, demonstrated high sequence identity (97.2% to 100%) with the reference material examined. In contrast, *T. panniferum* collections from the islands of Crete and Naxos growing in association with *Quercus* spp. (ACAMTub243, ACAMTub269, and ACAMTub385) showed some variation (97.3–97.5% sequence identity) when compared to three sequences available in international databases.

As regards the Excavatum clade, our analysis presents six to seven phylospecies; Greek specimens are distributed in four of them, with high support (98–100%, 1.00), i.e., *T. fulgens*, and another three are hereby provisionally named as *T.* aff. *excavatum* 1 (ACAMTub151, ACAMTub162), *T.* aff. *excavatum* 2 (ACAMTub028, ACAMTub316) and *T.* aff. *excavatum* 3 (ACAMTub168); each of them also includes collections of European origin.

In the Gennadii clade, the existence of six phylogenetic species was detected, with high support (98-100%, 1.00). Sequences from specimens studied in this work are grouped within two of them, one being the recently described *T. conchae* M. Romero & P. Alvarado. The other is one of the three phylospecies consisting of specimens identified as *T. gennadii*; hence, our two collections from the Attica area (ACAMTub050 and ACAMTub131) are grouped within a phylospecies provisionally labelled as *T.* aff. *gennadii*, which also includes specimens from Italy and Spain.

The Macrosporum clade is divided in two main well-supported groups (100%, 1.00). One of them corresponds to the *Tuber macrosporum* Vittad. species complex, and is further divided into three subgroups (98–100%, 0.99–1.00); all Greek *T. macrosporum* specimens originate from Central and Northern Greece and grow in association with a large range of angiosperms, and are grouped into one of these subgroups. In addition, one of the collections (ACAMTub455 from Paggaio Mt. in *Fagus sylvatica*) studied in this work corresponds to the European *T. monosporum* (Mattir.) Vizzini, on the basis of morphoanatomical features alone; no reference sequence for this species exists in the international databases.

As regards the Melanosporum clade, the majority of the respective specimens from Greece are grouped within the *T. brumale* species complex, while others (e.g., ACAMTub256) are identified as *T. melanosporum* (99.6–100% sequence identity with the species epitype, MZ423176), and originate from areas in the vicinity of truffle orchards.

Greek specimens in the Puberulum clade are grouped into at least six phylospecies with high support (96–100%, 1.00): *T. anniae* Vittad. (98.4% sequence identity with *T. anniae* holotype), *T. borchii* Vittad. s.l. (97.5–100% sequence identity with species lectotype MZ423174), *T. dryophilum* Tul. & C. Tul. (98.5–99.6%, when compared to reference sequence AF003917) and the *T. oligospermum* complex, including three phylogenetic species provisionally named *T*. aff. *oligospermum* 1, *T*. aff. *oligospermum* 2 and *T*. aff. *oligospermum* 3. The first and second phylospecies each include one collection studied in this work (ACAMTub075—Attica area and ACAMTub062—Fthiotida area, respectively), while the third includes five collections, all from Attica (Supplementary Table S1); this material is associated with *P. halepensis*, *P. pinea*, *Quercus coccifera*, *Q. ilex*, *Quercus* sp. and *Cistus monspeliensis*.

The presence of two species of the Regianum clade was detected in Greece, i.e., *T. magenctipunctatum* and *T. regianum*. Specimens of the former demonstrated high sequence identity (99.8–100%) with the species holotype, whereas the latter is represented by a single collection (ACAMTub226, Prespes area in *F. sylvatica*) which showed 98.0–100% sequence identity with the six *T. regianum* sequences deposited in GenBank.

Regarding the Rufum clade, Greek collections were grouped into seven phylospecies. Four of them correspond to known species: *T. aereum* (100% sequence identity with the holotype PP425915), *T. buendiae* Ant. Rodr. & Morte (97.8% sequence identity with the holotype MT006095), *T. nitidum* and *T. zambonelliae* (97.6–98.1% sequence identity with the holotype, MW632951). The other three form part of phylogenetically distinct groups (100%, 1.00) within the *T. rufum* species complex, which are provisionally named *T.* aff. *rufum* 1, *T.* aff. *rufum* 2 and *T.* aff. *rufum* 3. The first includes one collection from this study (ACAMTub251 from Olympos Mt.), and specimens identified as *T. rufum* from Italy and as *T. rufum* f. *ferrugineum* from Serbia; the second consists of one Greek collection (ACAMTub315 from the Prespes area) and one other specimen, labeled as *Tuber* sp. 57, from the USA. The third includes 23 collections from various regions of continental Greece (Supplementary Table S1), as well as specimens from Iran and Italy, identified as *T. rufum* f. *lucidum*, and from Estonia, identified as *Tuber* sp.

In particular, as concerns the Maculatum clade, a total of 51 ITS and 24 LSU sequences were analyzed; among these, 5 ITS and 5 LSU sequences were generated for the first time (Table 1). For the analysis of the two loci, the final alignment included 24 specimens and spanned 1406 nucleotides, comprising 544 nucleotides from the ITS and 862 from the LSU. The tree topologies obtained from the ML and BI analysis were similar, and the former was selected for the purposes of this study.

Taxa within the Maculatum clade present a worldwide distribution, by including collections originating from Europe, Asia, and North, Central and South America. European species are represented by *T. maculatum* Vittad., *T. foetidum* Vittad., *T. rapaeodorum* Tul. & C. Tul. and *T. lusitanicum* Ant. Rodr. & Muñoz-Mohedano. Most importantly, the phylogenetic analysis of the Maculatum clade revealed the presence of a new species to science, i.e., *Tuber leptodermum*, which forms a monophyletic group with 100% bootstrap support and a posterior probability of 1 (100%, 1.00) in individual ITS and LSU phylogenies and in concatenated ITS+LSU phylogenies (Figure 2 and Figure 3, Supplementary Figure S1).

In the ITS and in the concatenated ITS+LSU phylogenies, *T. leptodermum* was resolved as a sister to *T. foetidum* (83%, 0.99 and 78%, 0.97, respectively), while other phylogenetically related taxa were *T. hubeiense* L. Fan, *T. lusitanicum*, *T. maculatum*, *T. pseudomagnatum* L. Fan and *T. rapaeodorum*. Based on a megablast search of the INSDC (GenBank) nucleotide database, the closest hits using the ITS sequence of the *T. leptodermum* type material were collections identified as *T. foetidum* from Spain (MH703905, MT621657, MT621658) and Serbia (FM205704), presenting sequence identity values of 94.6–95.7%.

### 3.2. Taxonomy

**Figure 4:**
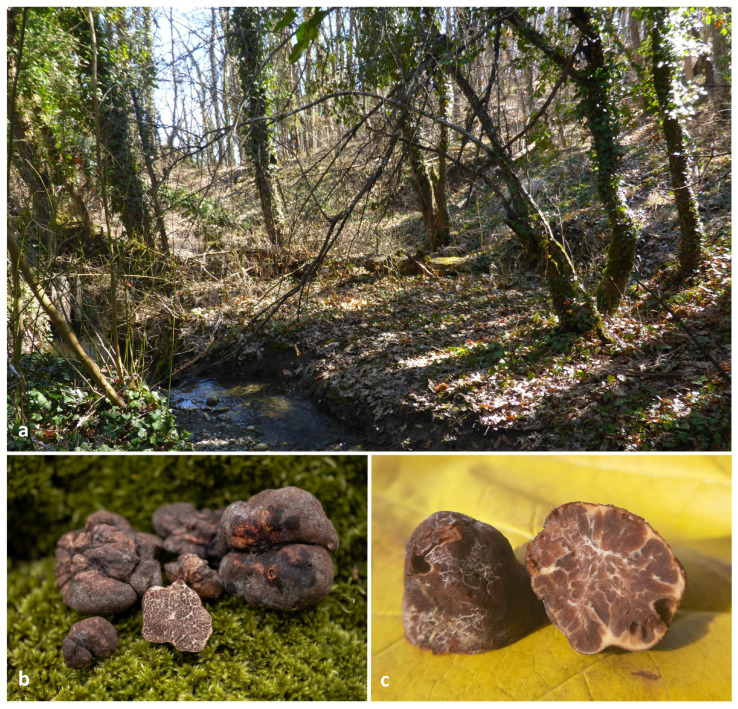
*Tuber leptodermum* sp. nov.: (**a**) representative biotope of occurrence (Grevena, collection code ACAMTub379); (**b**) mature ascomata in situ (holotype; ACAMTub475); (**c**) mature ascomata in situ (ACAMTub382).

**Figure 5:**
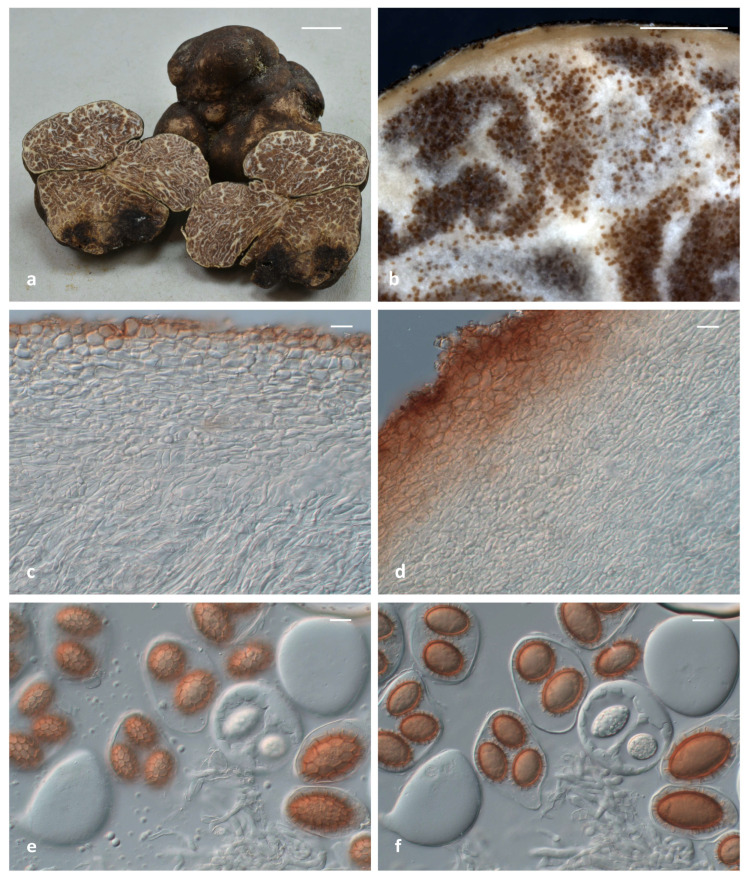
*Tuber leptodermum* sp. nov.: (**a**) mature ascomata ex situ (holotype; collection code ACAMTub475); (**b**) macroscopic details of the gleba and the peridium (holotype; ACAMTub475); (**c**) a peridium structure resembling a stonewall, colored only at the outer margin (ACAMTub380); (**d**) a peridium structure also colored towards the internal layer (ACAMTub379); (**e**) ascospore details featuring ornamentation of the polygonal meshes (ACAMTub380); (**f**) ascospore details with clear depiction of their outline and the size of the alveoli (ACAMTub380). Bars: (**a**) ascomata 1 cm; (**b**) gleba 1 mm; (**c**–**f**) microscopic features 20 μm.

*Tuber leptodermum* V. Daskalopoulos, G. Konstantinidis, N. Tsilis, E. Polemis & G.I. Zervakis, sp. nov. (Figure 4 and Figure 5).

MycoBank: MB858411

Etymology: The epithet *leptodermum* derives from the Greek words “λεπτό” (“lepto”, meaning thin) and “δέρμα” (“derma”, meaning skin), and refers to the thin peridium of the new species.

Diagnosis: Ascoma medium sized, dark-brown, peridum thin, up to 150 (170) μm, pseudoparenchymatous, composed of globose-to-angular cells, exoperidium easily scratched off to reveal a light-colored endoperidium; asci 1–4-spored (rather equally varied); ascospores ellipsoid, reticulate–alveolate, with broad meshes. It is found in riparian habitats dominated by *Populus* and/or *Salix* spp. *T. leptodermum* is a phylogenetically distinct species in the Maculatum clade, and represents a sister taxon to *T. foetidum*, as revealed by the ITS and the concatenated ITS+LSU phylogeny.

Holotype: GREECE. KOZANI: Municipality of Voio, Tsotyli greater area, elevation approx. 700 m, found in soil under *Populus alba*, 12 October 2024, coll. N. Tsilis (holotype ACAMTub475, designated here). GenBank: ITS = PQ877422; LSU = PQ881954.

Description: Ascomata hypogeous, 20–50 mm in diameter, generally irregular, tuberiform, knotty and lobed but also subglobose, with firm and solid texture, surface felty smooth to minutely verrucose to areolate in spots, color cigar-brown {16} to dark-umber {36} or darker-brown vinaceous {25} to purplish-chestnut {21} (Figure 5a). Peridium (50) 80–150 (170) µm thick, external layer (exoperidium) (15) 25–85 (130) µm thick, pseudoparenchymatous, of subglobose to irregularly angular cells (*textura globulosa-angularis*), dark-brick {20} in color, without any protruding cystidioid hyphae, cells (8.5) 11–18.5 (29) × (6.5) 7.5–13.5 (16.5) µm; internal layer (endoperidium) (50) 70–110 (125) µm thick, hyaline, with relatively smaller cells in comparison with the external layer and progressively more elongated towards the gleba (Figure 5b–d). Gleba firm, solid, and whitish at first, becoming fawn {29} to umber {18} or date-brown {24} at maturity, ornamented with numerous branching white veins (Figure 5a,b). Odor intense, penetrating, reminiscent of gas mixed with a somewhat spicy aromatic scent, like horseradish (*Armoracia rusticana*). Taste mild and sweetish, occasionally with a mustard-like aftertaste. Asci excluding stalk (70.5) 80–109 (122.5) × (47) 58–83.5 (945) µm, Av = 94.6 × 72.3 µm (n = 100), irregularly subglobose, broadly ellipsoid or pyriform, generally without, occasionally with a short stalk measuring 33–52 × 9–18.5 µm, with 1–4 ascospores per ascus (1-spored 24%, 2-spored 37%, 3-spored 27%, 4-spored 12%, n = 500), walls approx. 1 μm thick (Figure 5e,f). *Ascospores* (23.0) 28.9–53.6 (61.7) × (17.8) 22.7–36.9 (42.5) µm, Av: 40.4 × 28.8, Q = (1.01) 1.23–1.57 (1.93), Qav: 1.40 (n = 400), excluding ornamentation, cinnamon-to-rusty brown at maturity, generally broadly ellipsoid-to-ellipsoid, but also sometimes subglobose, ovoid or even oblong, and reticulate–alveolate, with broad meshes, (2.4) 3.6–5.9 (10.4) µm in height; meshes regular, closed and regularly polygonal (5-6 sides), 3-5 (6) lengthwise (3 = 15%, 4 = 43%, 5 = 33%, 6 = 9%, n = 100) (Figure 5e,f). Ascospores from one-spored asci (39.8) 44.7–58.7 (61.7) × (26.1) 31.6–39.8 (42.5) µm, Av = 52 × 35.7 µm; Q = (1.2) 1.3–1.6 (1.8), Qav = 1.5 (n = 100); two-spored asci (30.6) 36.5–47.6 (53.2) × (23.5) 25.5–33.2 (40.2) µm, Av = 42.6 × 29.5 µm; Q = (1) 1.3–1.6 (1.9), Qav = 1.5 (n = 100); three-spored asci (24.5) 28.5–40.4 (47.2) × (19.4) 22.3–29.4 (33.3) µm, Av = 34.6 × 26 µm; Q = (1.1) 1.2–1.5 (1.6), Qav = 1.3 (n = 100); four-spored asci (23) 27.5–38 (43.4) × (17.8) 21.7–27.6 (29.3) µm, Av = 32.5 × 24.1 µm; Q = (1) 1.2–1.5 (1.8), Qav = 1.4 (n = 100).

Distribution and ecology: *Tuber leptodermum* grows in relatively humid, deciduous forests or stands of *Populus*, *Quercus*, *Carpinus*, *Corylus* and/or *Salix*, predominantly near water streams and lakes, in habitats where *T. macrosporum* and (more often) *T. magnatum* also appear (Figure 4). The ascomata were found from October to November, at the Kozani and Grevena prefectures in NW Greece.

Additional specimens examined: GREECE. KOZANI: under *Populus*, *Quercus*, *Carpinus* and *Corylus*, 25 November 2021, coll. P. Kladopoulou (ACAMTub359; GenBank: ITS = PP918908; LSU = PP905688); GREECE. KOZANI: under trees of the genera *Populus* (*P. alba* and *P. tremula*) and *Quercus*, 28 November 2021, coll. N. Tsilis, GK13815 (ACAMTub382; GenBank: ITS = PP918911; LSU = PP905691); GREECE. GREVENA: under *Populus* (*P. alba, P. nigra* and *P. tremula*), *Quercus*, *Carpinus*, *Corylus* and *Salix*, 7 November 2021, coll. I. Kalampoukas, GK13481 (ACAMTub379; ITS = PP918909; LSU = PP905689); GREECE. GREVENA: under *Quercus* and *Salix*, 17 November 2021, coll. I. Kalampoukas GK13509 (ACAMTub380; ITS = PP918910; LSU = PP905690).

Notes: *T. leptodermum* sp. nov. is distinct from other related species of the Maculatum clade, i.e., *T. maculatum*, *T. rapaeodorum*, *T. lusitanicum*, *T. hubeiense*, *T. pseudomagnatum* and *T. foetidum*, as demonstrated in the ITS and LSU, and in the concatenated ITS+LSU phylogenies (Figure 2 and Figure 3, Supplementary Figure S1). As regards morphology, *T. leptodermum* produces medium-sized (20–50 mm diam), dark-brown ascomata with a thin (up to 150 (170) μm) pseudoparenchymatous peridium, a fawn-to-umber or date-brown gleba when mature, and cinnamon-to-rusty brown ascospores with broad meshes; mature ascomata have an intense and penetrating odor reminiscent of gas mixed with a somewhat spicy aromatic scent, like horseradish. It is mostly found in riparian habitats with predominantly *Populus* spp. Comparisons of such features with those of the aforementioned phylogenetically related taxa reveal several differences in the size and color of ascomata, in the thickness (mainly) and/or structure of the peridium, in the presence of hair-like hyphae on the peridium surface, in the gleba color and/or in the shape, color and ornamentation of the ascospores. Especially as regards *T. foetidum*, which is the closest phylogenetic taxon to *T. leptodermum*, it is morphologically very similar to *T. leptodermum*, but it is distinguished from the latter by the sparse presence of very short hair-like hyphae on the peridium surface, a thicker (200–350 µm) peridium and a fetid odor [12,13].

**Figure 6:**
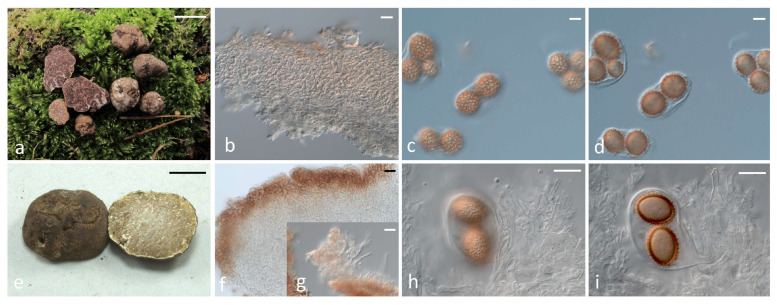

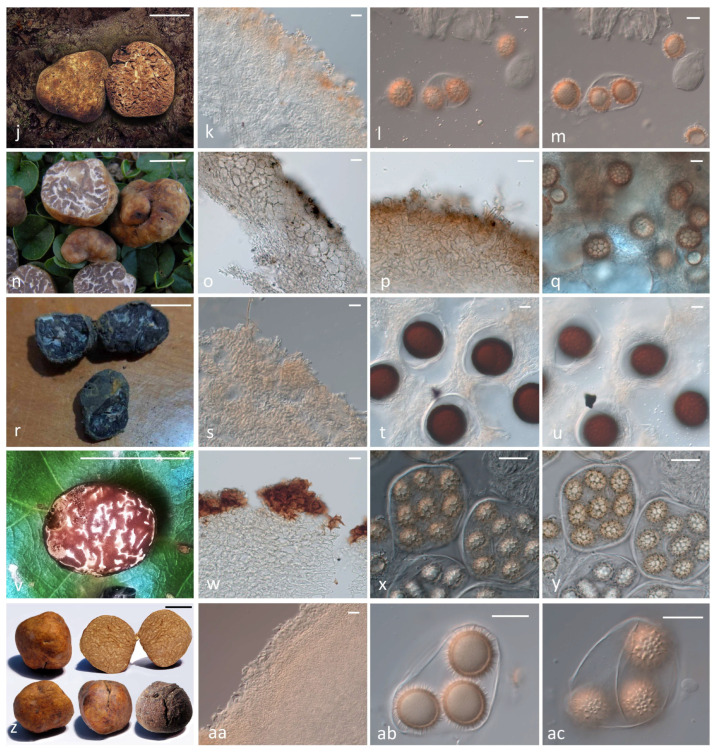
*Tuber* species corresponding to first records for the Greek mycobiota: (**a**–**d**) *T. anniae* ascomata, peridium structure and ascospores; (**e**–**i**) *T. buendiae* ascomata, peridium structure, dermatocystidia and ascospores; (**j**–**m**) *T. conchae* ascomata, peridium structure and ascospores; (**n**–**q**) *T. dryophilum* ascomata, peridium structure, peridium with dermatocystidia and ascospores; (**r**–**u**) *T. monosporum* ascomata, peridium structure and ascospores; (**v**–**y**) *T. regianum* ascomata, peridium structure and ascospores, (**z**–**ac**); *T. zambonelliae* ascomata, peridium structure and ascospores. Bars: ascomata 1 cm; microscopic features 20 μm.

As regards the seven *Tuber* species which are reported for the first time in Greece, a summary of the key morphoanatomic features of the respective collections, together with details about their distribution and ecology, are included in Supplementary Table S2. In addition, representative photos of their ascomata and microscopic characters are provided in Figure 6.

## 4. Discussion

The new truffle species, *T. leptodermum*, produces medium-sized (20–50 mm diam), dark-brown truffles which exhibit distinct morphological features, i.e. a felty smooth or sparsely warty, thin peridium (up to 150 (170) µm), pseudoparenchymatic, composed of globose-to-angular cells, and an exoperidium which is easily scratched off to reveal a light-colored endoperidium. In addition, the asci contain one to four reticulate–alveolate, ellipsoid ascospores with broad meshes. It was found to occur in riparian habitats, mainly under *Populus* and *Salix* spp., in autumn. *T. leptodermum* is a phylogenetically distinct species of the Maculatum clade, and is most closely related to *T. foetidum*, and then to *T. hubeiense*, *T. maculatum*, *T. rapaeodorum*, *T. pseudomagnatum* and *T. lusitanicum*, on the basis of the phylogenetic study performed.

When *T. leptodermum* was compared to the aforementioned species, the following differences were noted: *T. hubeiense* grows under *Pinus armandii* by forming small-sized (5–9 mm) whitish ascomata with a peridium of 180–250 µm, and yellow–brown-to-golden-brown ascospores [64]; *T. lusitanicum* also produces small-sized (5–20 mm) ascomata that are white at first, then pale-yellowish, sometimes with a reddish tinge, and are darker at maturity, with a thick peridium (300–500 µm) bearing sparse hair-like hyphae, an olive-brown-to-dark-brown gleba and yellowish-brown ascospores [59]; *T. maculatum* forms ascomata with a thick peridium (300–400 µm), prosenchymatous throughout, but often with scattered isodiametric cells or clusters of such cells, and with ellipsoid, light-golden-brown ascospores, ornamented with a regular reticulum 2–5 μm deep [91]; *T. pseudomagnatum* produces yellow–white ascomata with blackish gleba and small elliptic ascospores with a large meshed reticulum [63]; *T. rapaeodorum* produces small-sized (up to 25 mm), white-to-ochraceous or ochraceous-yellow (with dark-brown patches) ascomata with a thick peridium (250–600 µm), a snuff-brown-to-dark-purplish brown gleba and a horseradish- or radish-like odor [13].

In particular, as regards comparisons between *T. foetidum* and *T. leptodermum*, which appear to be the closest relatives (sequence identity values up to 95%), the two species demonstrate a high similarity in most of their morphoanatomical features; however, the former could be distinguished by the sparse presence of very short hair-like hyphae on the peridium surface, a thicker (200–350 µm) peridium and a fetid odor [12,13]. A phylogenetically distant species that could be confused with *T. leptodermum* is *T. macrosporum*; the latter also grows in similar (riparian) habitats, but it has is garlic-like odor and forms larger, darker colored ascospores with more meshes along the spore length.

**Figure 7:**
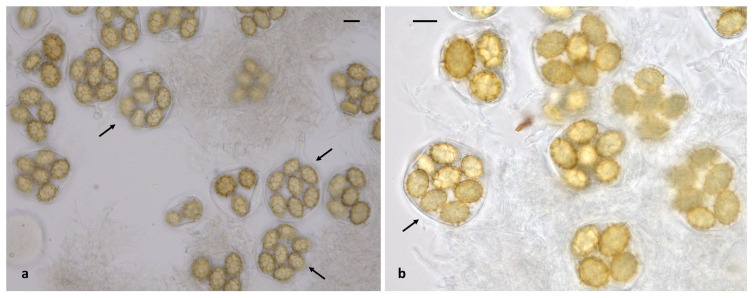
*Tuber aestivum* asci and ascospores; arrows indicate the presence of (**a**) 7-spored asci and (**b**) an 8-spored ascus. Bars: 20 μm.

Within the Aestivum clade, collections from our material were grouped into five species: *T. aestivum*, *T. bituminatum*, *T. magnatum*, *T. mesentericum* and *T. panniferum*; the presence of *T. panniferum* in Greece is molecularly confirmed for the first time. In addition, the occurrence of two other species of this clade, i.e., *T. pulchrosporum* and *T. suave*, has been previously recorded in various regions of the country and in the Attica area, respectively [27,29]. Especially as regards *T. aestivum*, a small number of seven-spored asci was noted in the majority of our collections, while a single eight-spored ascus was observed in one specimen only (Figure 7); hence, when seven collections of *T. aestivum* were examined, the presence of one-, two-, three-, four-, five-, six- and seven-spored asci was calculated at (percentages of total) 2.4%, 10.0%, 14.9%, 23.0%, 32.4%, 15.0% and 2.3%, respectively (n = 660). To the best of our knowledge, there is only one previous report on the presence of seven- and eight-spored asci in *T. aestivum* ascomata [92], despite the quite extensive pertinent literature. Noteworthy morphoanatomical variability was also detected in *T. mesentericum*; in measurements of ascospores in six collections, the quotient was Q = (1.01) 1.22 × 1.72 (2.17), with Qav = 1.47 (n = 613). These measurements differ substantially from the respective observations made by Leonardi et al. [27], suggesting the existence of more elongated ascospores in Greek *T. mesentericum* specimens. Furthermore, the latter is apparently limited to the northern part of the country, whereas *T. bituminatum* seems to be much more widespread and common (Supplementary Table S1).

In the phylogenetic analysis of our study, the Excavatum clade consisted of six highly supported phylospecies which included collections initially determined either as *T. excavatum* Vittad. or as *T. fulgens* Quél. (the presence of the latter is molecularly confirmed for the first time); however, none of them could be assigned with certainty to either one of these species, due to a lack of sequences from the respective type material. Most of the Greek specimens were grouped in *T.* aff. *excavatum* 3, which corresponds to “*T. excavatum* Clade IV” sensu Puliga et al. [47]; this phylospecies exhibits a large distribution throughout mainland Greece in coniferous, deciduous or mixed forests. Two additional phylospecies, i.e., *T.* aff. *excavatum* 1 and *T.* aff. *excavatum* 2, corresponding to “*T. excavatum* Clade I” and “*T. excavatum* Clade III” sensu Puliga et al. [47], respectively, include collections from the northern part of the country growing in association with broadleaved trees only. It should be noted that the existence of species within the Excavatum clade in Greece is molecularly confirmed for the first time.

As regards the Gennadii clade, *T. conchae* is reported for the first time in Greece. According to the original description (Romero and Alvarado in Crous et al. [48]), this species differs from *T. gennadii* because the latter “… has smaller (0.5–2 cm diam) and globose or subglobose ascomata (rarely wrinkled or lobulated), develops long cracks or locules in the gleba with age, and is probably associated to a different host plant (*Tuberaria guttata*)”. Interestingly, our collection’s features do not support the distinction of the two species based on these differences, since it consists of a small ascoma of ca. 2 cm diam, its gleba possesses locules and cracks, and it was found in the vicinity of *T. guttata* (Figure 6, Supplementary Table S2); still, the ITS sequence of the Greek collection exhibits 99.6% identity with the species holotype from Spain, leaving no doubts about its identity. It should be noted that this same *T. conchae* collection was previously identified as *T. gennadii* by using an LSU sequence only [26]. *T. gennadii* was described by Chatin [16] from material deriving from the Peloponnese, Greece. Recent collections from other regions of the country were examined and were identified as such together with specimens from Italy, Spain and north Morocco [23,26]; these correspond to three distinct phylospecies in our ITS tree (Figure 1). Our collections were provisionally labeled as *Tuber* aff. *gennadii*, and are grouped within one of the three aforementioned phylospecies by also including a specimen from Italy (BM1904, Genebank HM485361) which is considered to represent *T. gennadii* [43]. Therefore, the true phylogenetic position of this species will remain ambiguous until the holotype is sequenced.

The presence of *T. macrosporum* is molecularly confirmed for the first time in Greece. The respective collections are included—together with other sequences deriving from various areas of the Mediterranean Basin—in of one of the three phylogenetic groups formed with high support in the ITS tree (Figure 1); this particular group corresponds to *T. macrosporum* Clade I (sensu Benucci et al. [52]), while another group represents *T. macrosporum* Clade II, comprising sequences from France and Italy. The identity values between the sequences of *T. macrosporum* Clade I and II are less than 96.5%. The third group seems to be further separated, and consists of material originating from Iran [46]. Another species of the Macrosporum clade is *T. monosporum*, as revealed by the identity of a single collection from Greece which possesses the typical (and unique) morphoanatomical features of the species, and it was identified as such, although no pertinent sequences have been available until now in the international databases. This finding constitutes a new record for Greek mycobiota. It is worth mentioning that *T. monosporum* forms ascospores that differ considerably from those of other *Tuber* spp., since their surface has polygonal craters (like inward meshes) resembling a golf ball (Figure 6, Supplementary Table S2); therefore, it was originally placed in a distinct genus, i.e., *Paradoxa monospora* Mattir. However, Vizzini [93] transferred it to the genus *Tuber* as *T. monosporum* (Mattir.) Vizzini, and our phylogenetic analysis supports this placement.

As regards the Melanosporum clade in our phylogeny, it consists of six phylogroups, three corresponding to well-known species, i.e., *T. brumale* (Europe), *T. melanosporum* Vittad. (Europe) and *T. indicum* Cooke & Massee (China), whereas the rest represent the recently described *T. cryptobrumale* Merényi, T. Varga & Bratek, *T. petrophilum* Milenković, P. Jovan., Grebenc, Ivančević & Marković and *T. thracicum* Slavova, M. Leonardi, A. Paz, Assyov & Pacioni, all originating from the Balkan peninsula. Specimens from Greece are grouped either within the *T. brumale* species complex, or identified as *T. melanosporum*. Especially as regards the former, recent results indicate that the Melanosporum clade may have undergone evolutionary diversification, separating populations from eastern and southeastern Europe from those present in central and western Europe [8,9,70]. In addition, Mereneyi et al. [8] demonstrated that the “*T. brumale* aggregate” comprises three phylospecies corresponding to haplogroups I and II in Clade A and Clade B. The latter coincides with *T. cryptobrumale* [33], while haplogroup II exhibits a distribution which is limited to the wider Balkan region, including Greece, as our findings confirm; however, haplogroups I and II have yet to be formally described as distinct species. Concerning *T. melanosporum*, its occurrence in Greece has not been verified in areas other than those in close proximity to truffle orchards where this species is cultivated. Hence, and in the absence of other pertinent records in the country (or in neighboring regions), we believe that *T. melanosporum* is not a native species.

In the Puberulum clade, *T. dryophilum* is a relatively small, whitish and hairless truffle (or with very short hairs), with ellipsoid ascospores and a spore reticulum with very wide meshes, i.e., two to three along the spore length, as well as a plectenchymatous peridium structure [12]. However, the results of Lancellotti et al. [94] and our own observations on the respective material indicate the existence of a pseudoparenchymatic peridium with large angular cells and more rounded ascospores with 4–7 (8) meshes lengthwise (Figure 6; Supplementary Table S2). Thus, it is quite possible that specimens of *T. dryophilum* studied by Montecchi and Sarasini [12] correspond to another taxon from the reference collection of *T. dryophilum* (AF003917), as the latter is defined by Lancelotti et al. [94], and our specimens were compared with this. It is worth noting that ambiguities in discriminating the small whitish truffles of the Puberulum group s.l. (which includes the Maculatum and Gibbosum clades) are well documented, particularly by Lancellotti et al. [94], who stated that only 4 out of 44 sequences initially labeled as *T. dryophilum* were correctly identified.

As regards the rest of the species within the Puberulum clade, *T. anniae* W. Colgan & Trappe was first described in North America, growing in association with conifers. The respective Greek specimen is the first ever recorded in Greece, and it is morphoanatomically very similar to the holotype of *T. anniae* and other pertinent descriptions [11,95,96,97], by sharing the same pseudoparenchymatic peridium structure and the coniferous habitat (*Pinus sylvestris*, N. Greece). Furthermore, in our ITS phylogeny, *T. borchii* appears as a sister group to *T. anniae* (100%, 1.00; Figure 1). According to previous studies [32,72] this species consists of two “Haplotypes” with no apparent morphoanatomical or ecological differences between them. The majority of the Greek collections identified as *T. borchii* correspond to “Haplotype 1”, while three were grouped in “Haplotype 2” (Supplementary Table S1). As concerns *T. oligospermum*, the results of our phylogenetic analysis are in agreement with those of Alvarado et al. [26] by demonstrating that it corresponds to a complex of (at least) four distinct phylogenetic species with a predominantly Mediterranean distribution, three of which comprise specimens from Greece. To better distinguishing the latter, we have provisionally named them as *T*. aff. *oligospermum* 1, *T*. aff. *oligospermum* 2 and *T*. aff. *oligospermum* 3. Still, the phylogenetic grouping of *T. oligospermum* s.str. remains unclear, and to elucidate this issue, sequencing of the type specimen is required. Interestingly enough, our phylogenetic analysis shows that specimens previously reported as “*T. puberulum*” [26], i.e., GK4352 (JN392223) from Xanthi and GK5601 (JN392224) from Kozani, are grouped in *T. dryophilum*; thus, the presence of the latter in Greece is reported for the first time. In contrast, the existence of *T. puberulum* Berk. & Broome in the country has yet to be confirmed. This species was originally described from a collection found in the United Kingdom, and according to Lancellotti et al. [94], it appears almost exclusively in central and northern Europe, while it has not been recorded in Italy and in the Balkan peninsula.

The Regianum clade is composed of only three European species, i.e., *T. bernardini* Gori, *T. megentipuncatum* and *T. regianum*, demonstrating similar/overlapping morphoanatomic features, which makes it difficult to distinguish them by classical taxonomy only. Cseh et al. [28] reported that they could be discriminated by their relative ascospore size and volume, as well as by the average size of their ascospore meshes. However, our *T. regianum* collection possesses the *T. magenctipunctatum* ascospore pattern, both as regards size and volume. The presence of *T. regianum* is reported for the first time in Greece.

The Rufum clade includes some of the most taxonomically challenging groups, since it consists of many closely related (and several still undescribed) species exhibiting high phenotypic similarity, particularly within the *T. rufum* complex and in Mediterranean habitats [76,81,98]. In the frame of our work, the existence of *T. buendiae* and *T. zambonelliae* is recorded for the first time in Greece, thus expanding the known distribution of these species, since their presence had been reported in Spain and Morocco only. Moreover, sequences from Greek collections show a relatively high variation (up to 97.5% sequence identity values) compared to the holotypes of the two species. In addition, the existence of *T. nitidum* in Greece is molecularly confirmed for the first time, while *T. aereum* is also reported on Andros island, very close to the type locality (Naxos island; [30]). Apart from the four aforementioned taxa, our collections are grouped into three other well-supported phylospecies which were provisionally named *T.* aff. *rufum* 1, *T.* aff. *rufum* 2 and *T.* aff. *rufum* 3; the first two are recorded for the first time in Greece, while the existence of the third was also reported by Alvarado et al. [75]. Still, none of them could be assigned with certainty to *T. rufum*, due to absence of sequences from the respective type material. In the past, the presence of *T. rufum* was recorded in Greece as *T. rufum* var. *rufum* Pollini [20,21,99], as *T. rufum* var. *apiculatum* E. Fisch. [20] and as *T. rufum* f. *lucidum* (H. Bonnet) Montecchi & Lazzari [21], but it is not known yet to which of the aforementioned phylospecies these findings correspond to.

The findings of this study substantially increase our knowledge of truffle diversity, the range of associated plants, and their geographic distribution in Greece (Table 2). As a result, a total of 40 species (including 10 undescribed phylogenetic species) is now known to exist, as compared to 26 species which were previously reported, while the number of molecularly confirmed species has increased from 15 to 20.

**Table 2:** A summarized review of the known diversity of the genus *Tuber* in Greece, also including the findings of this study: taxa grouped per phylogenetic clade, associated plant(s), geographic distribution according to administrative regions of Greece, and pertinent references. Species whose presence has been confirmed by molecular approaches are presented in **bold**. Abbreviations for the administrative regions of Greece are as follows: East Macedonia and Thrace (EM & T), Western Macedonia (WM), Central Macedonia (CM), Epirus (Ep), Thessaly (Th), Western Greece (WG), Central Greece (CG), Attica (At), Peloponnese (P), Ionian Islands (I), North Aegean (NA), South Aegean (SA) and Crete (Cr). A map depicting the administrative regions of Greece is provided in the Supplementary Materials, Figure S2.

A/A	CLADE/Taxon	Associated Plant(s)	Distribution	References *
	AESTIVUM			
1	***T. aestivum*** Vittad. ^1^	*Abies, Pinus, Quercus, Corylus, Cistus*	EM & T, CM, WM, Ep, Th, WG, CG, At, P, SA	[18,20,21,41,100,101,102,103]; this study
2	***T. bituminatum*** Berk. & Broome	*Abies cephalonica, Abies borisii-regis, Pinus nigra, Quercus pubescens, Quercus coccifera, Quercus, Fagus sylvatica*	WM, CM, Th, CG, P	[27]; this study
3	***T. magnatum*** Picco	*Populus, Carpinus, Quercus, Salix, Tilia, Corylus, Pinus*	WM, CM, Th	[9,25,102]; this study
4	***T. mesentericum*** Vittad. ^1^	*F. sylvatica, Fagus, Corylus avelana, Corylus, Carpinus betulus, Carpinus, Quercus, Populus, Salix, Tilia, Pinus nigra, Pinus*	EM & T, WΜ, CM	[27,104]; this study
5	***T. panniferum*** Tul. & C. Tul.	*Q. pubescens, Q. coccifera, Quercus ilex, Quercus*	EM & T, CM, SA, Cr	[20,21,102]; this study
6	***T. pulchrosporum*** Konstantinidis, Tsampazis, Slavova, Nakkas, Polemis, Fryssouli & Zervakis	*Quercus, Corylus, Carpinus, Pinus*	EM & T, WM, Ep, Th, WG, At	[29]
7	***T. suave*** Pacioni & M. Leonardi	*Q. coccifera, Pinus halepensis*	At	[27]
	EXCAVATUM			
8	*T. excavatum* Vittad.	*Abies, Pinus, Quercus, Corylus, Tilia, Salix*	EM & T, CM, WM, Ep, P	[20,21,99]
9	***T.* aff. *excavatum* 1**	*Corylus*	WM	This study
10	***T.* aff. *excavatum* 2**	*F. sylvatica*	EM & T, WM	This study
11	***T.* aff. *excavatum* 3**	*A. cephalonica, Q. coccifera, Q. ilex, Quercus, F. sylvatica, Carpinus, P. halepensis*	EM & T, CM, WM, CG, At, P	This study
12	***T. fulgens*** Quél.	*Abies, F. sylvatica, Corylus, Carpinus, Quercus*	EM & T, CM, WM, Th	[21]; this study
	GENNADII			
13	*T. asa* Tul. & C. Tul. ^2^	*Cistus*	At	[23]
14	***T. conchae*** M. Romero & P. Alvarado ^3^	*Tuberaria guttata*	EM & T	This study
15	***T. gennadii*** (Chatin) Pat. ^4^	*Helianthemum, Cistaceae*	EM & T, CG, At, P	[16,21,23,26,105]
16	***T.* aff. *gennadii***	*T. guttata, P. halepensis, Pinus pinea, Q. coccifera*	At	This study
	MACROSPORUM			
17	***T. macrosporum*** Vittad.	*Corylus avelana, Corylus, Carpinus, Quercus, Salix, Tilia, Populus*	EM & T, WM, CM, Th	[24,102]; this study
18	***T. monosporum***(Mattir.) Vizzini	*F. sylvatica*	WM	This study
	MACULATUM			
19	***T. leptodermum*** Daskalopoulos, Konstantinidis, Tsilis, Polemis & Zervakis	*Populus alba, Populus tremula, Populus nigra, Quercus, Salix, Corylus, Carpinus*	WM	This study
	MELANOSPORUM			
20	***T. brumale*** Vittad. ^5^	*Quercus, Carpinus, Fagus*	EM & T, Ep, CM, WM, P	[8,20,21,33,104]; this study
21	***T. melanosporum*** Vittad. ^6^	*Quercus, Carpinus*	CM	[20,21,102,104]; this study
22	*T. cibarium* Corda			[17]
	PUBERULUM			
23	***T. anniae*** W. Colgan & Trappe	*Pinus sylvestris*	CM	This study
24	***T. borchii*** Vittad.	*Abies, Pinus, Quercus, Erica*	EM & T, CM, WM, Ep, Th, WG, CG, At, P, NA, SA	[20,21,26,99,102,103,104]; this study
25	***T. dryophilum*** Tul. & C. Tul.	*Quercus, Carpinus, Pinus*	EM & T, WM, Th, At, P	This study
26	***T.* aff*. oligospermum* 1**	*P. halepensis, P. pinea, Q. coccifera*	At	[26]; this study
27	***T.* aff*. oligospermum* 2**	*Quercus*	CG	[26]; this study
28	***T.* aff*. oligospermum* 3**	*P. halepensis, Q. ilex, C. monspeliensis*	At	[26]; this study
29	*T. puberulum* Berk. & Broome ^7^	*Quercus*	EM & T, WM	[20,26,104]
	REGIANUM			
30	***T. magentipunctatum*** Merényi, I. Nagy, Stielow & Bratek	*A. cephalonica, C. avelana*	WM, P	[28]
31	***T. regianum*** Montecchi & Lazzari	*F. sylvatica*	WM	This study
	RUFUM			
32	***T. aereum*** Polemis, Daskalopoulos & Zervakis	*Q. coccifera, Q. pubescens, Q. macrolepis*	SA	[30]
33	***T. buendiae*** Ant. Rodr. & Morte	*A. cephalonica*	CG	This study
34	*T. ferrugineum* Vittad.	*Abies, Corylus, Quercus*	CM, WM, Th, CG, At, P, NA	[21,99,106]
35	***T. nitidum*** Vittad. ^8^	*Quercus, Carpinus*	EM & T, Ep, Th, At	[20]; this study
36	*Tuber rufum* Pollini ^9^	*Abies, Quercus, Carpinus, Cistus*	EM & T, WM, CM, Th, At	[20,21,99]
37	***T.* aff. *rufum* 1**	*Populus, Carpinus, Salix, Tilia*	CM	This study
38	***T.* aff. *rufum* 2**	*F. sylvatica*	WM	This study
39	***T.* aff. *rufum* 3** ^10^	*A. cephalonica, Pinus nigra, Pinus, Q. coccifera, Quercus, F. sylvatica*	EM & T, WM, Ep, Th, CG, At, P	[75]; this study
40	***T. zambonelliae*** Ant. Rodr. & Morte	*P. halepensis, Q. ilex, Quercus, Carpinus*	WM, At	This study

^1^ Reported findings prior to 2021 could correspond to any one of the following species: *T. aestivum*, *T. mesentericum*, *T. bituminatum* and *T. suave.* ^2^ The collections initially identified as *T. asa* [23] were later assessed as *T. oligospermum* [26]. ^3^ The collection was initially identified as *Loculotuber gennadii* [21], and later as *T. gennadii* [26]. ^4^ The collections identified as *T. gennadii* by Alvarado et al. [26] correspond to more than one phylospecies, which also include *T.* aff. *gennadii.* ^5^ Reported as *T. brumale* or *T. brumale* f. *moschatum* in the pertinent literature. ^6^ The only molecularly confirmed record of this species that does not originate directly from a truffle orchard with cultivated *T. melanosporum* (but in the vicinity of one) derives from this study. ^7^ The collections examined by Alvarado et al. [26] were identified as *T. dryophilum* by this study. ^8^ Reported as *Tuber rufum* var. *nitidum* (Vittad.) Mont. & Lazzari by Diamandis and Perlerou [20]. ^9^ Reported either as *T. rufum*, *T. rufum* var. *rufum*, or *T. rufum* f. *lucidum* or *T. rufum* var. *apiculatum* in the pertinent literature. ^10^ Reported as *T. rufum* by Alvarado et al. [75]. * References [8,9,26,27,28,29,30,41,75] include molecularly identified specimens.

## 5. Conclusions

In the frame of the study, the presence of 31 phylospecies of the genus *Tuber* is reported in Greece. These correspond to one new species to science (i.e., *Tuber leptodermum*); to 20 well-described taxa, 7 of which (*T. anniae*, *T. buendiae, T. conchae*, *T. dryophilum*, *T. monosporum*, *T. regianum*, and *T. zambonelliae*) constitute first national records; and to 10 undescribed phylogenetic species within the *T. excavatum*, *T. gennadii*, *T. oligospermum* and *T. rufum* species complexes (the existence of 6 is revealed for the first time). Collections of the *T. excavatum* complex are grouped into three phylospecies, while those initially identified as *T. oligospermum* and *T. rufum* appear as members of three distinct clades in each one of the respective species complexes. Moreover, sequences from Greece labeled as *T. gennadii* correspond to two distinct phylospecies, while the presence of *T. puberulum* in Greece could not be confirmed, since all specimens previously identified as such were grouped within *T. dryophilum*. *T. melanosporum* is considered non-native to the country, and *T. bituminatum* seems to be much more common than the phylogenetically related and morphologically similar *T. mesentericum*. In addition, a sequence representing *T. monosporum* has been generated (and becomes available) for the first time, representing a collection deriving from Northern Greece. Our results, as well as the outcome of other pertinent studies, emphasize the need for epitypification (or neotypification) of several truffle species occurring in Europe. In addition, a combined effort of truffle taxonomists could focus on the description of cryptic species within the Rufum and Excavatum clades, and among the small whitish truffles (mainly in the Puberulum and Gennadii clades), where the corresponding collections demonstrate high levels of phenotypic similarity and considerable sequence variation.

## Data Availability

The data presented in the manuscript are available on request from the corresponding author. In addition, sequences generated by this study are deposited in GenBank, and phylogenetic trees and pertinent data are deposited in TreeBASE.

## Supplementary Materials

The following supporting information can be downloaded at https://www.mdpi.com/article/10.3390/jof11050358/s1: Figure S1: A maximum likelihood (ML) tree, based on LSU sequences, depicting the phylogeny of the Maculatum clade by including *Tuber leptodermum* sp. nov., which appears in bold typeface. *Choiromyces helanshanensis* is used as an outgroup. Bootstrap support (>70%) for ML and Bayesian posterior probabilities (>0.95) are indicated at the tree nodes; values of 100% and 1.00, respectively, are marked by asterisks. The use of sequences from type specimens is indicated by “T” placed as superscript after the respective GenBank accession number. Figure S2: A map depicting the administrative regions of Greece. Abbreviations (and the respective colors) are as follows: East Macedonia and Thrace (EM & T—in red), Western Macedonia (WM—in purple), Central Macedonia (CM—in green), Epirus (Ep—in blue), Thessaly (Th—in orange), Western Greece (WG—in yellow), Central Greece (CG—in red), Attica (At—in purple), Peloponnese (P—in blue), Ionian Islands (I—in magenta), North Aegean (NA—in yellow), South Aegean (SA—in green) and Crete (Cr—in orange). Table S1. Biological material of the genus *Tuber* deriving from Greece and examined in the frame of this study: phylogenetic clade, taxon, fungarium (collection) code and collector’s code, geographic origin, associated plant(s) and GenBank accession no. for ITS and LSU sequences. Collections used in the phylogenetic analyses are presented in bold typeface. Table S2: Detailed descriptions of *Tuber anniae*, *T. buendiae*, *T. conchae*, *T. dryophilum*, *T. monosporum*, *T. regianum* and *T. zambonelliae*, which constitute first records for the Greek mycobiota. The number of samples, as well as the numbers of all measurements, are shown in brackets (n).
