# Supplementary material for: The Diversity of the Genus Tuber in Greece—A New Species to Science in the Maculatum Clade and Seven First National Records
Source: J Fungi (Basel). 2025 May 5;11(5):358. doi: 10.3390/jof11050358 (PMC12112760; doi:10.3390/jof11050358)
Supplement: Supplementary file 1 [file jof-11-00358-s001.zip › jof-3577608-supplementary figures.pdf]

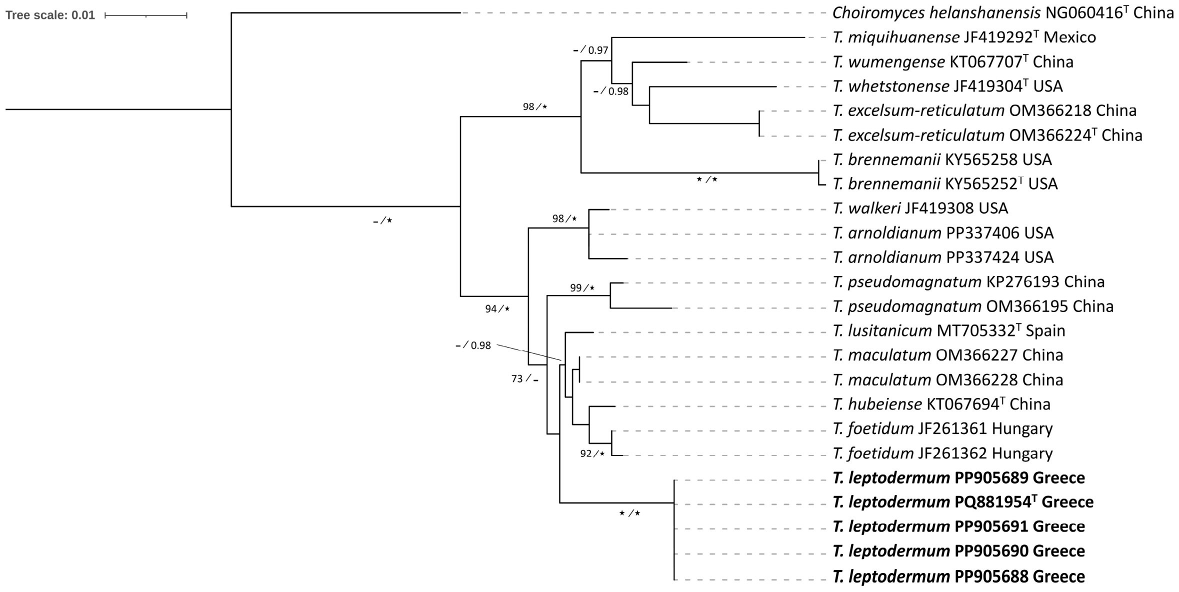

Figure S1: A maximum likelihood (ML) tree, based on LSU sequences, depicting the phylogeny of the Maculatum clade by including *Tuber leptodermum* sp. nov., which appears in bold typeface. *Choiromyces helanshanensis* is used as an outgroup. Bootstrap support (>70%) for ML and Bayesian posterior probabilities (>0.95) are indicated at the tree nodes; values of 100% and 1.00, respectively, are marked by asterisks. The use of sequences from type specimens is indicated by "T" placed as superscript after the respective GenBank accession number.

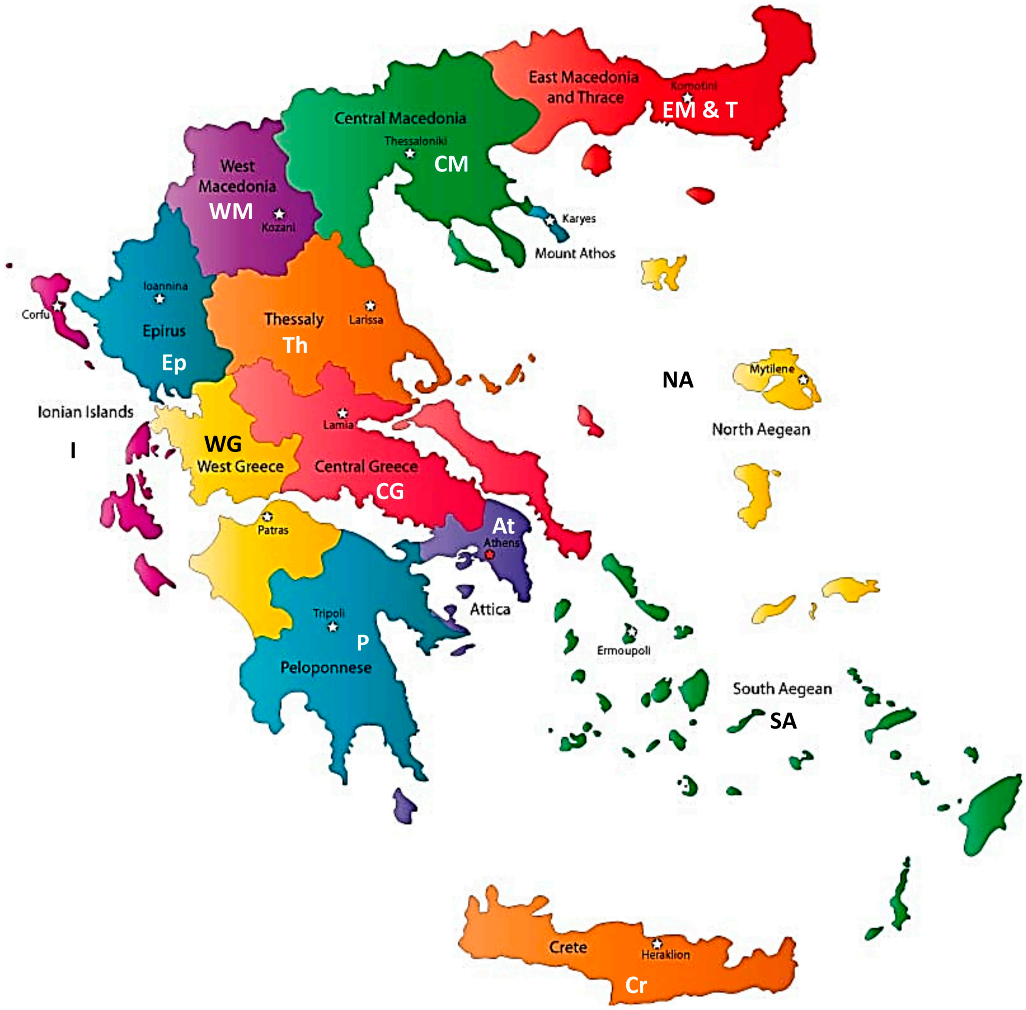

Figure S2: A map depicting the administrative regions of Greece. Abbreviations (and the respective colors) are as follows: East Macedonia and Thrace (EM & T—in red), Western Macedonia (WM—in purple), Central Macedonia (CM—in green), Epirus (Ep—in blue), Thessaly (Th—in orange), Western Greece (WG—in yellow), Central Greece (CG—in red), Attica (At—in purple), Peloponnese (P—in blue), Ionian Islands (I—in magenta), North Aegean (NA—in yellow), South Aegean (SA—in green) and Crete (Cr—in orange).
