# Supplementary material for: The Diversity of the Genus Tuber in Greece—A New Species to Science in the Maculatum Clade and Seven First National Records
Source: J Fungi (Basel). 2025 May 5;11(5):358. doi: 10.3390/jof11050358 (PMC12112760; doi:10.3390/jof11050358)
Supplement: Supplementary file 1 [file jof-11-00358-s001.zip › jof-3577608-supplementary tables.pdf]

SUPPLEMENTARY MATERIAL

Table S1. Biological material of the genus *Tuber* deriving from Greece and examined in the frame of this study: phylogenetic clade and taxon, fungarium (collection) code and collector's code, geographic origin and corresponding administrative region, associated plant(s), and GenBank accession no. for ITS and LSU sequences. Collections used in the phylogenetic analyses are presented in **bold** typeface. Abbreviations for the administrative regions of Greece are as follows: East Macedonia and Thrace [EM & T], Western Macedonia [WM], Central Macedonia [CM], Epirus [Ep], Thessaly [Th], Western Greece [WG], Central Greece [CG], Attica [At], Peloponnese [P], Ionian Islands [I], North Aegean [NA], South Aegean [SA], Crete [Cr]. A map of the administrative regions of Greece is provided in Supplementary Material, Figure S2.

A/A	CLADE / Taxon	Fungarium code	Collector's code	Origin [Administrative Region]	Associated plant(s)	ITS	LSU
	AESTIVUM						
1	*T. aestivum*	ACAMTub003		Arkadia: Mainalon Mt. [P]	*Abies cephalonica*	PP918654	
2	*T. aestivum*	ACAMTub004		Arkadia: Mainalon Mt. [P]	*A. cephalonica*	PP918655	
3	*T. aestivum*	ACAMTub007	GK 2681	Kastoria: Gavros [WM]	*Fagus sylvatica, Carpinus, Quercus*	PP918656	
4	*T. aestivum*	ACAMTub072	VK 1233	Attica: Katsimidi [At]	*Quercus ilex, Pinus halepensis*	PP918657	
5	*T. aestivum*	ACAMTub081		Arkadia: Mainalon Mt. [P]	*A. cephalonica*	PP918658	
6	*T. aestivum*	ACAMTub091		Fthiotida: Domokos [CG]	*Quercus frainetto*	PP918659	
7	*T. aestivum*	ACAMTub095		Arkadia: Parnonas Mt. [P]	*A. cephalonica, Quercus coccifera*	PP918660	
8	*T. aestivum*	ACAMTub096		Arkadia: Parnonas Mt. [P]	*A. cephalonica, Q. coccifera*	PP918661	
9	*T. aestivum*	ACAMTub098		Arkadia: Parnonas Mt. [P]	*A. cephalonica, Q. coccifera*	PP918662	
10	*T. aestivum*	ACAMTub102		Arkadia: Parnonas Mt. [P]	*A. cephalonica, Q. coccifera*	PP918663	
11	*T. aestivum*	ACAMTub147	MG 1712	Peloponnese [P]	Unknown	PP918665	
12	*T. aestivum*	ACAMTub172	VK 5273	Serres [CM]	*F. sylvatica*	PP918666	
13	*T. aestivum*	ACAMTub174	VK 5613	Attica: Parnitha Mt. [At]	*A. cephalonica*	PP918667	
14	*T. aestivum*	ACAMTub194		Trikala: Gorgogyri [Th]	*Carpinus*	PP918669	
15	*T. aestivum*	ACAMTub221		Pieria: Olympos Mt. [CM]	*Q. coccifera, Carpinus betulus, Pinus*	PP918670	
16	*T. aestivum*	ACAMTub222		Trikala: Kalampaka [Th]	*Q. coccifera*	PP918671	
17	*T. aestivum*	ACAMTub236		Trikala: Kalampaka [Th]	*Abies borisii-regis, Quercus*	PP918672	
18	*T. aestivum*	ACAMTub247		Pieria: Olympos Mt. [CM]	*Populus, Carpinus, Salix, Tilia, Quercus, Corylus avelana*	PP918673	
19	*T. aestivum*	ACAMTub268		Naxos Isl. [SA]	*Q. coccifera*	PP918674	
20	*T. aestivum*	ACAMTub270		Pieria: Olympos Mt. [CM]	*Quercus, Carpinus*	PP918675	

21	***T. aestivum***	**ACAMTub271**	**Ioannina** [Ep]	***Quercus, Q. coccifera***	**PP918676**
22	*T. aestivum*	ACAMTub274	Kastoria [WM]	*Quercus*	PP918677
23	*T. aestivum*	ACAMTub279	Veroia [CM]	*Pinus nigra, F. sylvatica*	PP918678
24	*T. aestivum*	ACAMTub280	Veroia [CM]	*P. nigra, F. sylvatica*	PP918679
25	*T. aestivum*	ACAMTub282	Veroia [CM]	*P. nigra, F. sylvatica*	PP918681
26	*T. aestivum*	ACAMTub283	Veroia [CM]	*P. nigra, F. sylvatica*	PP918682
27	*T. aestivum*	ACAMTub293	Veroia [CM]	*P. nigra, F. sylvatica*	PP918683
28	*T. aestivum*	ACAMTub294	Veroia [CM]	*P. nigra, F. sylvatica*	PP918684
29	*T. aestivum*	ACAMTub295	Veroia [CM]	*P. nigra, F. sylvatica*	PP918685
30	*T. aestivum*	ACAMTub296	Veroia [CM]	*P. nigra, F. sylvatica*	PP918686
31	*T. aestivum*	ACAMTub297	Veroia [CM]	*P. nigra, F. sylvatica*	PP918687
32	*T. aestivum*	ACAMTub344	Arkadia: Mainalon Mt. [P]	*A. cephalonica*	PP918689
33	*T. aestivum*	ACAMTub348	Fokida: Parnassos Mt. [CG]	*A. cephalonica*	PP918690
34	*T. aestivum*	ACAMTub353	Lakonia: Taygetos Mt. [P]	*A. cephalonica*	PP918691
35	*T. aestivum*	ACAMTub363	Arkadia: Mainalon Mt. [P]	*A. cephalonica*	PP918692
36	*T. aestivum*	ACAMTub365	Arkadia: Mainalon Mt. [P]	*A. cephalonica*	PP918693
37	***T. aestivum***	**ACAMTub376**	**Xanthi** [EM & T]	**Unknown**	**PP918694**
38	*T. aestivum*	ACAMTub391	Grevena [WM]	Unknown	PP918695
39	*T. aestivum*	ACAMTub392	Grevena [WM]	Unknown	PP918696
40	*T. aestivum*	ACAMTub393	Grevena [WM]	Unknown	PP918697
41	*T. aestivum*	ACAMTub394	Grevena [WM]	Unknown	PP918698
42	*T. aestivum*	ACAMTub395	Grevena [WM]	Unknown	PP918699
43	*T. aestivum*	ACAMTub405	Pieria: Olympos Mt. [CM]	Unknown	PP918700
44	*T. aestivum*	ACAMTub409	Fthiotida: Kallidromo Mt. [CG]	*A. cephalonica*	PP918702
45	*T. aestivum*	ACAMTub411	Fthiotida: Kallidromo Mt. [CG]	*A. cephalonica*	PP918703
46	*T. aestivum*	ACAMTub423	Arkadia: Mainalon Mt. [P]	*A. cephalonica, P. nigra, Acer pseudoplatanus*	PP918704
47	*T. aestivum*	ACAMTub123B	Arkadia: Mainalon Mt. [P]	*A. cephalonica*	PP918755
48	*T. aestivum*	ACAMTub338B	Veroia [CM]	*P. nigra, F. sylvatica*	PP918886
49	*T. bituminatum*	ACAMTub123A	Arkadia: Mainalon Mt. [P]	*A. cephalonica*	PP918664
50	*T. bituminatum*	ACAMTub085	Arkadia: Mainalon Mt. [P]	*A. cephalonica*	PP918747
51	*T. bituminatum*	ACAMTub086	Arkadia: Mainalon Mt. [P]	*A. cephalonica*	PP918748
52	*T. bituminatum*	ACAMTub097	Arkadia: Parnonas Mt. [P]	*A. cephalonica, Q. coccifera*	PP918749
53	*T. bituminatum*	ACAMTub101	Arkadia: Parnonas Mt. [P]	*A. cephalonica, Q. coccifera*	PP918751
54	*T. bituminatum*	ACAMTub103	Arkadia: Parnonas Mt. [P]	*A. cephalonica, Q. coccifera*	PP918752
55	*T. bituminatum*	ACAMTub119	Arkadia: Parnonas Mt. [P]	*A. cephalonica, Q. coccifera*	PP918753

56	*T. bituminatum*	ACAMTub122B		Arkadia: Mainalon Mt. [P]	*A. cephalonica*	PP918754
57	*T. bituminatum*	ACAMTub140	MG 1705	Peloponnese [P]	Unknown	PP918756
58	*T. bituminatum*	ACAMTub175		Arkadia [P]	Unknown	PP918757
59	*T. bituminatum*	ACAMTub238		Trikala: Kalampaka [Th]	*A. borisii-regis, Quercus*	PP918758
60	*T. bituminatum*	ACAMTub304		Veroia [CM]	*P. nigra, F. sylvatica*	PP918759
61	*T. bituminatum*	ACAMTub305		Veroia [CM]	*P. nigra, F. sylvatica*	PP918760
62	*T. bituminatum*	ACAMTub306		Veroia [CM]	*P. nigra, F. sylvatica*	PP918761
63	*T. bituminatum*	ACAMTub307		Veroia [CM]	*P. nigra, F. sylvatica*	PP918762
64	*T. bituminatum*	ACAMTub308		Veroia [CM]	*P. nigra, F. sylvatica*	PP918763
65	***T. bituminatum***	**ACAMTub310**		**Florina: Prespes** [WM]	***F. sylvatica***	**PP918764**
66	*T. bituminatum*	ACAMTub323		Florina: Prespes [WM]	*F. sylvatica*	PP918765
67	*T. bituminatum*	ACAMTub345		Arkadia: Mainalon Mt. [P]	*A. cephalonica*	PP918766
68	*T. bituminatum*	ACAMTub346		Fokida: Parnassos Mt. [CG]	*A. cephalonica*	PP918767
69	*T. bituminatum*	ACAMTub349		Fokida: Parnassos Mt. [CG]	*A. cephalonica*	PP918769
70	*T. bituminatum*	ACAMTub374		Grevena: Perivoli [WM]	*P. nigra*	PP918770
71	*T. bituminatum*	ACAMTub398	VK 6800	Arkadia [P]	*A. cephalonica*	PP918771
72	*T. bituminatum*	ACAMTub399	VK 6801	Arkadia [P]	*A. cephalonica*	PP918772
73	*T. bituminatum*	ACAMTub400	VK 6802	Arkadia [P]	*A. cephalonica*	PP918773
74	*T. bituminatum*	ACAMTub401	VK 6803	Arkadia [P]	*A. cephalonica*	PP918774
75	*T. bituminatum*	ACAMTub402	VK 6804	Arkadia [P]	*A. cephalonica*	PP918775
76	*T. bituminatum*	ACAMTub403	VK 6805	Arkadia [P]	*A. cephalonica*	PP918776
77	*T. bituminatum*	ACAMTub417		Arkadia: Mainalon Mt. [P]	*A. cephalonica, P. nigra, A. pseudoplatanus*	PP918777
78	*T. bituminatum*	ACAMTub418		Arkadia: Mainalon Mt. [P]	*A. cephalonica, P. nigra, A. pseudoplatanus*	PP918778
79	*T. bituminatum*	ACAMTub419		Arkadia: Mainalon Mt. [P]	*A. cephalonica, P. nigra, A. pseudoplatanus*	PP918779
80	*T. bituminatum*	ACAMTub420		Arkadia: Mainalon Mt. [P]	*A. cephalonica, P. nigra, A. pseudoplatanus*	PP918780
81	***T. bituminatum***	**ACAMTub421**		**Arkadia: Mainalon Mt.** [P]	***A. cephalonica, P. nigra, A. pseudoplatanus***	**PP918781**
82	*T. magnatum*	ACAMTub053	GK 5202	Kastoria [WM]	*Salix*	PP918867
83	***T. magnatum***	**ACAMTub055**		**Larissa: Olympos Mt.** [Th]	**Unknown**	**PP918868**
84	*T. magnatum*	ACAMTub115		Grevena [WM]	*Quercus*	PP918869
85	***T. magnatum***	**ACAMTub156**	**NT 223**	**Grevena** [WM]	***Quercus*, *Populus***	**PP918870**
86	*T. magnatum*	ACAMTub355		Grevena [WM]	*Populus*, *Carpinus*, *Quercus*, *C. avelana*	PP918871
87	*T. magnatum*	ACAMTub356		Grevena [WM]	*Populus*, *Carpinus*, *Quercus*, *C. avelana*	PP918872
88	*T. magnatum*	ACAMTub357		Grevena [WM]	*Populus*, *Carpinus*, *Quercus*, *C. avelana*	PP918873

89	*T. magnatum*	ACAMTub388		Grevena [WM]	Unknown	PP918874
90	*T. magnatum*	ACAMTub389		Grevena [WM]	Unknown	PP918875
91	*T. magnatum*	ACAMTub404		Pieria: Olympos Mt. [CM]	Unknown	PP918876
92	***T. mesentericum***	**ACAMTub111**		**Kilkis: Paiko Mt.** [CM]	***Corylus, Carpinus, Quercus***	**PP918880**
93	*T. mesentericum*	ACAMTub113		Kilkis: Paiko Mt. [CM]	*Corylus, Carpinus, Quercus*	PP918881
94	*T. mesentericum*	ACAMTub173	VK 5271	Serres [CM]	*F. sylvatica*	PP918882
95	*T. mesentericum*	ACAMTub234		Pieria: Olympos Mt. [CM]	*C. avelana, Populus, Tilia, Quercus*	PP918883
96	*T. mesentericum*	ACAMTub338A		Veroia [CM]	*P. nigra, F. sylvatica*	PP918688
97	*T. mesentericum*	ACAMTub249		Pieria: Olympos Mt. [CM]	*Populus*, *Carpinus*, *Salix, Tilia*, *Quercus*, *C. avelana*	PP918884
98	***T. mesentericum***	**ACAMTub250**		**Pieria: Olympos Mt.** [CM]	***Populus*, *Carpinus*, *Salix, Tilia*, *Quercus*, *C. avelana***	**PP918885**
99	*T. panniferum*	ACAMTub243		Naxos Isl. [SA]	*Q. coccifera*	PP918895
100	***T. panniferum***	**ACAMTub269**		**Naxos Isl.** [SA]	***Q. coccifera***	**PP918896**
101	***T. panniferum***	**ACAMTub385**		**Crete Isl.: Rethymno** [Cr]	***Quercus pubescens, Q. coccifera, Q. ilex***	**PP918897**
	EXCAVATUM					
102	***T.* aff. *excavatum* 1**	**ACAMTub151**	**NT 202**	**Grevena** [WM]	***Corylus***	**PP918825**
103	***T.* aff. *excavatum* 1**	**ACAMTub162**	**NT 215**	**Kastoria: Kato Pteria** [WM]	***Corylus***	**PP918826**
104	***T.* aff. *excavatum* 2**	**ACAMTub028**	**GK 2682**	**Xanthi** [EM & T]	***F. sylvatica***	**PP918828**
105	***T.* aff. *excavatum* 2**	**ACAMTub316**		**Florina: Prespes** [WM]	***F. sylvatica***	**PP918829**
106	*T.* aff. *excavatum* 3	ACAMTub083		Arkadia: Mainalon Mt. [P]	*A. cephalonica*	PP918830
107	*T.* aff. *excavatum* 3	ACAMTub092		Arkadia: Parnonas Mt. [P]	*A. cephalonica, Q. coccifera*	PP918831
108	*T.* aff. *excavatum* 3	ACAMTub104		Arkadia: Parnonas Mt. [P]	*A. cephalonica, Q. coccifera*	PP918832
109	*T.* aff. *excavatum* 3	ACAMTub105		Arkadia: Parnonas Mt. [P]	*A. cephalonica, Q. coccifera*	PP918833
110	*T.* aff. *excavatum* 3	ACAMTub106		Arkadia: Parnonas Mt. [P]	*A. cephalonica, Q. coccifera*	PP918834
111	*T.* aff. *excavatum* 3	ACAMTub116		Arkadia: Parnonas Mt. [P]	*A. cephalonica, Q. coccifera*	PP918835
112	*T.* aff. *excavatum* 3	ACAMTub129	VK 5258	Attica: Parnitha Mt. [At]	*A. cephalonica*	PP918836
113	*T.* aff. *excavatum* 3	ACAMTub141	MG 1706	Peloponnese [P]	Unknown	PP918837
114	*T.* aff. *excavatum* 3	ACAMTub142	MG 1707	Peloponnese [P]	Unknown	PP918838
115	*T.* aff. *excavatum* 3	ACAMTub143	MG 1708	Peloponnese [P]	Unknown	PP918839
116	*T.* aff. *excavatum* 3	ACAMTub145	MG 1710	Peloponnese [P]	Unknown	PP918840
117	***T.* aff. *excavatum* 3**	**ACAMTub168**	**VK 4785**	**Arkadia: Mainalon Mt.** [P]	***A. cephalonica***	**PP918841**
118	*T.* aff. *excavatum* 3	ACAMTub322a		Florina: Prespes [WM]	*F. sylvatica*	PP918842
119	*T.* aff. *excavatum* 3	ACAMTub341		Arkadia: Mainalon Mt. [P]	*A. cephalonica*	PP918843
120	*T.* aff. *excavatum* 3	ACAMTub413		Fthiotida: Kallidromo Mt. [CG]	*A. cephalonica*	PP918844
121	*T.* aff. *excavatum* 3	ACAMTub031	GK 5357	Kavala: Elafochori [EM & T]	*Quercus*	PP918845
122	*T.* aff. *excavatum* 3	ACAMTub032	GK 5386	Kastoria: Korestia [WM]	*Carpinus, Quercus*	PP918846
123	*T.* aff. *excavatum* 3	ACAMTub033	VK 1246	Attica: Katsimidi [At]	*P. halepensis, Q. ilex*	PP918847

124	*T.* aff. *excavatum* 3	ACAMTub034		Attica: Katsimidi [At]	*P. halepensis, Q. ilex*	PP918848	
125	*T.* aff. *excavatum* 3	ACAMTub035	VK 856	Attica: Katsimidi [At]	*P. halepensis, Q. ilex*	PP918849	
126	*T.* aff. *excavatum* 3	ACAMTub157	NT 225	Grevena [WM]	*Corylus*	PP918850	
127	***T.* aff. *excavatum* 3**	**ACAMTub320a**		**Florina: Prespes** [WM]	***F. sylvatica***	**PP918851**	
128	*T. fulgens*	ACAMTub108	NT 216	Kilkis: Paiko Mt. [CM]	*Corylus, Carpinus, Quercus*	PP918852	
129	***T. fulgens***	**ACAMTub170**	**VK 5270**	**Serres** [CM]	***F. sylvatica***	**PP918853**	
130	***T. fulgens***	**ACAMTub312**		**Florina: Prespes** [WM]	***F. sylvatica***	**PP918854**	
131	*T. fulgens*	ACAMTub325		Florina: Prespes [WM]	*F. sylvatica*	PP918855	
	GENNADII						
132	*T.* aff. *gennadii*	ACAMTub048	VK 843	Attica: Schinias [At]	*Tuberaria guttata, P. halepensis, Pinus pinea, Q. coccifera*	PP918856	
133	***T.* aff. *gennadii***	**ACAMTub050**		**Attica: Schinias** [At]	***T. guttata, P. halepensis, P. pinea, Q. coccifera***	**PP918857**	
134	***T.* aff. *gennadii***	**ACAMTub131**	**VK 4816**	**Attica: Schinias** [At]	***T. guttata, P. halepensis, P. pinea, Q. coccifera, C. monspeliensis***	**PP918858**	
135	***T. conchae***	**ACAMTub046**	**GK 3035**	**Xanthi** [EM & T]	***T. guttata***	**PP918816**	
	MACROSPORUM						
136	***T. macrosporum***	**ACAMTub052**	**GK 5899**	**Kastoria** [WM]	**Broadleaves**	**PP918859**	
137	***T. macrosporum***	**ACAMTub110**		**Kilkis: Paiko Mt.** [CM]	***Corylus, Carpinus, Quercus***	**PP918860**	
138	*T. macrosporum*	ACAMTub235		Pieria: Olympos Mt. [CM]	*C. avelana, Populus, Tilia, Quercus*	PP918861	
139	***T. macrosporum***	**ACAMTub252**		**Larissa: Olympos Mt.** [Th]	***Populus, Carpinus, Salix, Tilia, Quercus, C. avelana***	**PP918862**	
140	*T. macrosporum*	ACAMTub254		Pieria: Olympos Mt. [CM]	*C. avelana, Populus, Tilia, Quercus*	PP918863	
141	*T. macrosporum*	ACAMTub309		Kozani [WM]	*Populus, Carpinus, Quercus, C. avelana*	PP918864	
142	***T. monosporum***	**ACAMTub455**	**VK 7160**	**Kavala: Paggaio Mt.** [EM & T]	***F. sylvatica***	**PP918907**	PP892729
	MACULATUM						
143	***T. leptodermum***	**ACAMTub359**		**Kozani** [WM]	***Populus* spp.*, Carpinus, Quercus, C. avelana***	**PP918908**	**PP905688**
144	***T. leptodermum***	**ACAMTub379**	**GK 13481**	**Grevena: Elatos** [WM]	***Populus alba, Populus tremula, Populus nigra, Quercus, Salix, Corylus, Carpinus***	**PP918909**	**PP905689**
145	***T. leptodermum***	**ACAMTub380**	**GK 13509**	**Grevena** [WM]	***Quercus, Salix***	**PP918910**	**PP905690**
146	***T. leptodermum***	**ACAMTub382**	**GK 13815**	**Kozani** [WM]	***P. alba, P. tremula, Quercus***	**PP918911**	**PP905691**
147	***T. leptodermum***	**ACAMTub475**		**Kozani** [WM]	***P. alba***	**PQ877422**	**PQ881954**
	MELANOSPORUM						
148	*T. brumale*	ACAMTub018	GK 2677	Kastoria: Gavros [WM]	*F. sylvatica, Carpinus, Quercus*	OP850806	
149	***T. brumale***	**ACAMTub159**		**Preveza** [Ep]	***Quercus, Q. coccifera***	**OP850807**	
150	*T. brumale*	ACAMTub112		Kilkis: Paiko Mt. [CM]	*Corylus, Carpinus, Quercus*	OP850808	
151	*T. brumale*	ACAMTub019	GK 5380	Kastoria: Korestia [WM]	*Carpinus, Quercus*	OP850809	

152	*T. brumale*	ACAMTub150	NT 201	Grevena [WM]	*Quercus*, *Carpinus*	OP850810
**153**	***T. brumale***	**ACAMTub233**	**GK 9159**	**Ioannina** [Ep]	**Unknown**	**PP918804**
154	*T. brumale*	ACAMTub339		Pieria: Olympos Mt. [CM]	*Carpinus, Quercus, C. avelana*	PP918805
155	*T. brumale*	ACAMTub360		Pieria: Olympos Mt. [CM]	*Populus*, *Carpinus*, *Quercus* , *C. avelana*	PP918806
156	*T. brumale*	ACAMTub429		Kastoria [WM]	Unknown	PP918807
157	*T. brumale*	ACAMTub430		Kastoria [WM]	Unknown	PP918808
158	*T. brumale*	ACAMTub431		Kastoria [WM]	Unknown	PP918809
159	*T. brumale*	ACAMTub432		Ileia: Lambeia [WG]	*C. orientalis*	PP918810
160	*T. brumale*	ACAMTub433		Ileia: Lambeia [WG]	*C. orientalis*	PP918811
161	*T. brumale*	ACAMTub434		Ileia: Lambeia [WG]	*C. orientalis*	PP918812
162	*T. brumale*	ACAMTub435		Ileia: Lambeia [WG]	*C. orientalis*	PP918813
**163**	***T. brumale***	**ACAMTub456**	**VK 7161**	**Attica: Parnitha Mt.** [At]	***A. cephalonica***	**PP918814**
164	*T. melanosporum*	ACAMTub056	GK 2821	Pieria: Ritini [CM]	Truffle orchard with broadleaved trees	PP918877
**165**	***T. melanosporum***	**ACAMTub256**		**Pieria: Olympos Mt.** [CM]	***Quercus*, *Q. ilex*, *Carpinus***	**PP918878**
166	*T. melanosporum*	ACAMTub459		Korinthia: Feneos [P]	Truffle orchard with broadleaved trees	PP918879
	PUBERULUM					
**167**	***T.* aff. *oligospermum* 1**	**ACAMTub075**	**VK 722**	**Attica: Schinias** [At]	***P. halepensis, P. pinea, Q. coccifera***	**PP918887**
**168**	***T.* aff. *oligospermum* 2**	**ACAMTub062**	**GK 2953**	**Fthiotida: Kallidromo Mt.** [CG]	***Quercus***	**PP918889**
169	*T.* aff. *oligospermum* 3	ACAMTub063	VK 4475	Attica: Rafina [At]	*P. halepensis*	PP918890
170	*T.* aff. *oligospermum* 3	ACAMTub076	VK 806	Attica: Artemida [At]	*Cistus monspeliensis, P. halepensis*	PP918891
171	*T.* aff. *oligospermum* 3	ACAMTub080	VK 852	Attica: Katsimidi [At]	*P. halepensis, Q. ilex*	PP918892
**172**	***T.* aff. *oligospermum* 3**	**ACAMTub133**	**VK 5207**	**Attica: Rafina** [At]	***P. halepensis***	**PP918893**
173	*T.* aff. *oligospermum* 3	ACAMTub165	VK 5571	Attica: Rafina [At]	*P. halepensis*	PP918894
**174**	***T. anniae***	**ACAMTub024**	**GK 5099**	**Pella: Voras Mt.** [CM]	***P. sylvestris***	**PP918746**
175	*T. borchii* (haplotype 1)	ACAMTub469		Andros Isl. [SA]	*Q. pubescens*	PQ336173
176	*T. borchii* (haplotype 1)	ACAMTub013	GK 3054	Trikala: Aspropotamos [Th]	*Quercus, Q. coccifera*	PP918782
177	*T. borchii* (haplotype 1)	ACAMTub014	GK 5516	Kozani: Protochori [WM]	*Quercus, Q. coccifera*	PP918783
178	*T. borchii* (haplotype 1)	ACAMTub023	GK 5417	Rodos Isl.: Archagelos [SA]	Unknown	PP918784
179	*T. borchii* (haplotype 1)	ACAMTub073	VK 1271	Attica: Katsimidi [At]	*Q. ilex, P. halepensis*	PP918785
**180**	***T. borchii* (haplotype 1)**	**ACAMTub135**	**VK 5585**	**Attica: Katsimidi** [At]	***P. halepensis, Q. ilex***	**PP918787**
181	*T. borchii* (haplotype 1)	ACAMTub169	VK 4046	Attica: N. Makri [At]	*Erica, P. halepensis*	PP918788
182	*T. borchii* (haplotype 1)	ACAMTub180		Evia [CG]	*Pinus*	PP918789
183	*T. borchii* (haplotype 1)	ACAMTub185		Achaia [WG]	*P. halepensis, P. pinea*	PP918790
184	*T. borchii* (haplotype 1)	ACAMTub206		Trikala: Pyli [Th]	*Pinus, Cistus*	PP918791
**185**	***T. borchii* (haplotype 1)**	**ACAMTub334**		**Kozani** [WM]	***Carpinus, Quercus, C. avelana***	**PP918792**
186	*T. borchii* (haplotype 1)	ACAMTub367A		Peloponnese [P]	Unknown	PP918793

187	*T. borchii* (haplotype 1)	ACAMTub368		Pieria [CM]	Unknown	PP918794
188	*T. borchii* (haplotype 1)	ACAMTub369		Kozani [WM]	*P. halepensis*	PP918795
189	***T. borchii* (haplotype 1)**	**ACAMTub371A**		**Chalkidiki** [CM]	***Pinus***	**PP918796**
190	*T. borchii* (haplotype 1)	ACAMTub372		Thessaloniki [CM]	*Pinus*	PP918797
191	*T. borchii* (haplotype 1)	ACAMTub410		Fthiotida: Kallidromo Mt. [CG]	*A. cephalonica*	PP918798
192	*T. borchii* (haplotype 1)	ACAMTub439		Ileia: Lambeia [WG]	*Carpinus orientalis*	PP918799
193	*T. borchii* (haplotype 1)	ACAMTub446		Lesvos Isl. [NA]	*P. halepensis, P. pinea, Q. coccifera*	PP918800
194	*T. borchii* (haplotype 1)	ACAMTub120B		Arkadia: Mainalon Mt. [P]	*A. cephalonica*	PP918801
195	***T. borchii* (haplotype 2)**	**ACAMTub120A**		**Arkadia: Mainalon Mt.** [P]	***A. cephalonica***	**PP918786**
196	***T. borchii* (haplotype 2)**	**ACAMTub207**		**Trikala: Elati** [Th]	***A. borisii-regis***	**PP918802**
197	***T. borchii* (haplotype 2)**	**ACAMTub377**	**GK 4972**	**Pieria: Olympos Mt.** [CM]	***A. borissi-regis, F. sylvatica***	**PP918803**
198	*T. dryophillum*	ACAMTub015	VK 1312	Attica: Katsimidi [At]	*P. halepensis, Q. ilex*	PP918817
199	*T. dryophillum*	ACAMTub026	GK 4857	Kozani: Pelekanos [WM]	*Quercus*	PP918818
200	***T. dryophillum***	**ACAMTub043**	**GK 2918**	**Xanthi** [EM & T]	***C. avelana, Pinus***	**PP918819**
201	*T. dryophillum*	ACAMTub074	VK 4482	Attica: Katsimidi [At]	*Q. ilex, P. halepensis*	PP918820
202	***T. dryophillum***	**ACAMTub134**	**VK 5588**	**Attica: Katsimidi** [At]	***P. halepensis, Q. ilex***	**PP918821**
203	***T. dryophillum***	**ACAMTub182**		**Korinthia: Goura** [P]	***Q. pubescens***	**PP918822**
204	*T. dryophillum*	ACAMTub192		Trikala: Nea Zoi [Th]	*Carpinus*	PP918823
205	***T. dryophillum***	**ACAMTub204**		**Trikala: Megali Kerasia** [Th]	***Quercus***	**PP918824**
	REGIANUM					
206	***T. magenctipunctatum***	**ACAMTub126**		**Arkadia: Mainalon Mt.** [P]	***A. cephalonica***	**PP918865**
207	***T. magenctipunctatum***	**ACAMTub381**	**GK 13809**	**Florina: Prespes** [WM]	***C. avelana***	**PP918866**
208	***T. regianum***	**ACAMTub226**	**VK5490**	**Florina: Prespes** [WM]	***F. sylvatica***	**PP918898**
	RUFUM					
209	***T. aereum***	**ACAMTub467**		**Andros Isl.** [SA]	***Q. macrolepis***	**PQ336045**
210	*T. aereum*	ACAMTub470		Andros Isl. [SA]	*Q. pubescens*	PQ336161
211	***T.* aff. *rufum* 1**	**ACAMTub251**		**Pieria: Olympos Mt.** [CM]	***Populus*, *Carpinus*, *Salix, Tilia*, *Quercus*, *C. avelana***	**PP918918**
212	*T. nitidum*	ACAMTub038	GK 3524	Thessaloniki: Efkarpia [CM]	*Q. coccifera*	PP918706
213	***T. nitidum***	**ACAMTub039**	**GK 3542**	**Magnesia: Agioi Theodoroi** [Th]	***Quercus***	**PP918707**
214	***T. nitidum***	**ACAMTub375**		**Xanthi** [EM & T]	**Unknown**	**PP918708**
215	***T.* aff. *rufum* 2**	**ACAMTub315**		**Florina: Prespes** [WM]	***F. sylvatica***	**PP918919**
216	***T. buendiae***	**ACAMTub350**		**Fokida: Parnassos Mt.** [CG]	***A. cephalonica***	**PP918815**
217	***T.* aff. *rufum* 3**	**ACAMTub067**	**GK 2012**	**Xanthi** [EM & T]	***Pinus, Quercus***	**PP918709**
218	***T.* aff. *rufum* 3**	**ACAMTub230**	**GK 11677**	**Ioannina: Metsovo** [Ep]	**Unknown**	**PP918710**
219	*T.* aff. *rufum* 3	ACAMTub351		Fokida: Parnassos Mt. [CG]	*A. cephalonica*	PP918711

220	*T.* aff. *rufum* 3	ACAMTub412		Fthiotida: Loutra Ypatis [CG]	*A. cephalonica*	PP918712
221	*T.* aff. *rufum* 3	ACAMTub155	NT 217	Trikala: Kalampaka [Th]	*Quercus*	PP918713
222	***T.* aff. *rufum* 3**	**ACAMTub317**		**Florina: Prespes** [WM]	***F. sylvatica***	**PP918714**
223	*T.* aff. *rufum* 3	ACAMTub324a		Florina: Prespes [WM]	*F. sylvatica*	PP918715
224	*T.* aff. *rufum* 3	ACAMTub460		Korinthia: Feneos [P]	Truffle orchard with broadleaved trees	PP918716
225	*T.* aff. *rufum* 3	ACAMTub020		Arkadia: Mainalon Mt. [P]	*A. cephalonica*	PP918717
226	*T.* aff. *rufum* 3	ACAMTub094		Arkadia: Parnonas Mt. [P]	*A. cephalonica, Q. coccifera*	PP918718
227	*T.* aff. *rufum* 3	ACAMTub107		Arkadia: Parnonas Mt. [P]	*A. cephalonica, Q. coccifera*	PP918719
228	*T.* aff. *rufum* 3	ACAMTub144	MG 1709	Peloponnese [P]	Unknown	PP918720
229	*T.* aff. *rufum* 3	ACAMTub158		Korinthia: Mougosto [P]	*Quercus*, *A. cephalonica*	PP918721
230	*T.* aff. *rufum* 3	ACAMTub186		Arkadia: Mainalon Mt. [P]	*A. cephalonica, Q. coccifera*	PP918722
231	*T.* aff. *rufum* 3	ACAMTub227	MG 320	Peloponnese [P]	*A. cephalonica*	PP918723
232	*T.* aff. *rufum* 3	ACAMTub228	MG 346	Peloponnese [P]	*A. cephalonica*	PP918724
233	*T.* aff. *rufum* 3	ACAMTub229	MG 319	Peloponnese [P]	*A. cephalonica*	PP918725
234	*T.* aff. *rufum* 3	ACAMTub343		Arkadia: Mainalon Mt. [P]	*A. cephalonica*	PP918726
235	***T.* aff. *rufum* 3**	**ACAMTub425**		**Arkadia: Mainalon Mt.** [P]	***A. cephalonica, P. nigra, A. pseudoplatanus***	**PP918727**
236	*T.* aff. *rufum* 3	ACAMTub426		Arkadia: Mainalon Mt. [P]	*A. cephalonica, P. nigra, A. pseudoplatanus*	PP918728
237	*T.* aff. *rufum* 3	ACAMTub427		Arkadia: Mainalon Mt. [P]	*A. cephalonica, P. nigra, A. pseudoplatanus*	PP918729
238	*T.* aff. *rufum* 3	ACAMTub428		Arkadia: Mainalon Mt. [P]	*A. cephalonica, P. nigra, A. pseudoplatanus*	PP918730
239	*T.* aff. *rufum* 3	ACAMTub121		Arkadia: Mainalon Mt. [P]	*A. cephalonica*	PP918731
240	*T. zambonelliae*	ACAMTub017	VK 851	Attica: Katsimidi [At]	*P. halepensis, Q. ilex*	PP918920
241	***T. zambonelliae***	**ACAMTub040**	**GK 5389**	**Kastoria: Korestia** [WM]	***Carpinus, Quercus***	**PP918921**
242	***T. zambonelliae***	**ACAMTub079**	**VK 838**	**Attica: Katsimidi** [At]	***Q. ilex, P. halepensis***	**PP918922**

SUPPLEMENTARY MATERIAL

Table S2. Detailed descriptions of *Tuber anniae*, *T. buendiae*, *T. conchae*, *T. dryophilum*, *T. monosporum*, *T. regianum* and *T. zambonelliae* which constitute first records for the Greek mycobiota. The number of samples, as well as the numbers of all measurements, are shown in brackets (n).

	T. anniae (n=1)	***T. buendiae*** (n=1)	***T. conchae*** (n=1)	***T. dryophilum*** (n=10)
Ascomata				
Diameter	8 x 15 mm	20 x 25 mm	18 x 22 mm	5–50 mm
Shape	subglobose, irregularly globose to slightly tuberiform or lobed, with shallow furrows	globose	irregularly globose, tuberiform	irregularly globose, tuberiform to reniform, sometimes lobed or intensively knotty
Surface	smooth or minutely pubescent along furrows	veruccose-areolate with flattened warts, and furrows	smooth or minutely pubescent as immature, becoming rough with hollows when it is mature	smooth or minutely pubescent especially along furrows
Color	argent-whitish [A,1], clay pink [30] with somewhat saffron tints, fawn [29] to cigar brown [16]	brown to dark brown, snuff brown [17] to cigar brown [16]	yellowish buff [52] with clay buff [32] or snuff brown [17] spots	at first argent-whitish [A,1] to beige whitish [C3], turning to buff [52] and finally fulvous-buff [12-52] with rusty tawny tints [14]
Peridium				
Width (n=number of measurements)	(31) 50.7–116.1 (149.8) μm, thick in total, Av: 86.7 μm (n=71)	(149) 192.6–603.6 (823) μm thick in total, Av= 372.2 μm, (n=70)	(68.2) 102.6–190.8 (235.9) μm thick in total, Av= 154.7 μm, (n=96)	(59.5) 93.6–136.3 (168.8) μm, Av: 111.3 μm, (n=90)
General Texture	composed of pseudoparenchymatic, roundish, polygonal or puzzle-like compressed cells.	composed of an internal layer of hyaline, agglutinated, interwoven hyphae (intricate texture), and an external layer with an intermediate state between very densely agglutinated plectenchymatous texture towards to pseudoparenchymatic small and irregularly globose to subangular cells	composed of an external suprapellis with hyphal tips, cystidia and vesicules, erratically arranged, and an underlying pellis densely arranged as a plectenchyma	composed of pseudoparenchymatic, roundish to polygonal large sized cells, with cylindrical or clavate, septate dermatocystidia; non differentiated to external and internal layer with different structure, only sometimes with different color
External layer dimensions	(8.5) 12.1–27.4 (35) μm, Av=19.9 (n=67)	(28.1) 35.9–68.4 (86.1) μm, Av=53.8 (n=70)	(15.5) 24.4–51.6 (77.6) μm, Av 36.3 (n=92)	

External layer texture	of the same texture, with rare septate dermatocystidia towards the furrows	externally of the outer layer, agglutinated hyaline hyphae and scattered dermatocystidia, especially along the furrows	as previously explained	
External layer color	faintly pigmented orange brownish or hyaline	distinctly pigmented brick– brown	hyaline or faintly pigmented orange brownish, vesicules are mainly pigmented hazel brown	faintly pigmented orange brownish or hyaline
Cell dimensions (of pseudoparenchymatic external layer)	(7.7) 9–14.9 (19.3) × (4.7) 6.4–11.9 (13.6) µm, Av = 12 × 8.7 µm, n = 50			(9.3) 12.3–23.5 (34.7) × (6.6) 9.3–18 (23.8) µm, Av = 17.7 × 13.7 µm (n = 268)
External elements	cystidia, cylindrical or clavate, (14) 16.6–23.9 (31.5) × (2.7) 2.9–4.7 (5.6) µm, (n=7)	cystidia, septate, clavate or cylindrical	cystidia straight or curved, septate, solitary or connate, variedly shaped, often inflated at the middles or with capitate edges, resembling tibiiform cystidia, or clavate with acute tips to lageniform, sometimes forming an irregular trichoderm along with hyphal tips, (10.2) 13.8–29.2 (36.7) × (2.8) 3.4–6.3 (7) µm (n = 40) vesicules as inflated parts of variably shaped cystidia, or solitary as spherical cystidioid elements, almost always internally filled with orange–hazel brown pigment. (3.1) 4.1–8.1 (9.7) × (3) 3.3–7 (8.5) µm (n = 24)	cystidia (11.4) 17.3–30.3 (44.3) × (3.2) 3.6–6.1 (6.9) µm, (n = 42)
Gleba				
Texture	firm, solid	firm, solid	loose and fluffy when mature because of scattered irregular hollows (locules like *T. gennadii*) parallel to irregularly arranged veins	firm, solid
Color	whitish at first, becoming clay pink [30], purplish date [22] to bay [19] at maturity, marbled with numerous thin white veins	whitish at first, hazel [27] at maturity	when mature same colored like surface, ochre-grayish ranging from yellowish buff [52] with clay buff [32] to snuff brown [17]	whitish at first [A, D], becoming smoke gray [34], vinaceous grey [80] and finally drab [33] at maturity
Odor	pleasant, truffle like	not recorded	not recorded	weak odor, sometimes reminiscent of garlic or acetylene

Asci				
Dimensions (excluding stalk)	(74.3) 79.3–99.2 (107.7) × (46.7) 51.8–80 (85.9) µm, Av = 89.7 × 67.5 µm (n=46)	(37) 54.5–77.7 (96) × (28.7) 38.1–50.3 (59.7) µm, Av = 65.6 × 44.6 µm (n = 66)	(61.3) 62.1–116.1 (120.5) × (43.8) 44.5–78.6 (83.2) µm, Av = 86.6 × 59.7 µm (n = 13)	(76) 91.2–115.8 (125.8) × (58.4) 73.7–96.6 (107.5) µm, Av = 103.4 × 84 µm, (n = 80)
Shape	generally pyriform to subglobose, but often also ovate, ellipsoid or reniform, without or with a very short stalk	mainly pyriform with a long stalk, ranging from irregularly subglobose to nearly fusiform	generally pyriform or irregularly ellipsoid, with a stalk	generally ovoid, subglobose or globose, but often also ovate, without stalk
Stalk dimensions	20–25 x 5.5–8 µm (n=2)	(30.7) 30.9–40.7 (52.2) × (4.9) 5.4–6.9 (7.5) µm (n=10)	(21.2) 21.21–33.5 × 11.1–13.38 (13.4) µm (n=3)	
Rate of ascospores per asci	1–4 ascospores per asci, 1-spored 10%, 2-spored 46%, 3-spored 37%, 4-spored 7% (n=100)	1–4 ascospores per asci, 1-spored 5%, 2-spored 29%, 3-spored 33%, 4-spored 33% (n=200)	no records are available since the specimen was overmature	1 to 4 ascospores per asci, with ratios 1-spored 20%, 2-spored 43%, 3-spored 30%, 4-spored 7% (n=100)
Ascospores				
Total dimensions (excluding ornamentation)	(25.6) 31.9–53.7 (63) × (22) 28.0–44.7 (55.4) µm, Av=39.49 x 34.39; Q = (1.01) 1.06–1.23(1.41), Qav=1.15 (n=273)	(15.9) 20.9–30.31 (37.9) × (13.4) 16.2–23.3 (28.2) µm, Av = 65.6 × 44.6 µm; Q = (1) 1.18–1.47 (1.72), Qav=1.34 (n=217)	(24.8) 28.8–38.5 (50) × (21) 26.3–35.7 (41.9) µm, Av = 33.3 × 30.7 µm; Q = 1–1.2 (1.7), Qav = 1.1, (n = 132)	(28.3) 34.8–51.2 (60.4) × (24.7) 30–43.5 (49.2) µm, Av = 42.8 × 36.5 µm; Q = (1) 1.1–1.3 (1.5), Qav = 1.2, (n = 121)
Total spore volume (only for Regianum Clade truffles–0.523 x W2 x L–excluding ornamentation; Merenyi et al 2017)				
1-spored asci	(49.6) 53.3–57.9 (63) × (40.2) 44.1–47.1 (55.4) µm, Av = 55.3 × 45.9 µm; Q = (1.1) 1.2–1.3 (1.4), Qav = 1.2 (n = 36)	(26.5) 28.8–37 (37.9) × (21.1) 22–27.1 (28.2) µm, Av = 33.6 × 24.4 µm; Q = 1.2–1.5 (1.6), Qav = 1.4 (n=23)		
2-spored asci	(30.3) 35.9–45.7 (49.6) × (27.4) 31–39 (42.9) µm, Av = 41.2 × 35.6 µm; Q = (1) 1.1–1.2 (1.4), Qav = 1.2, (n = 100)	(19.3) 23.8–30 (32.7) × (14.2) 18–23.3 (24.4) µm, Av = 27.2 × 20.5 µm; Q = (1.1) 1.2–1.4 (1.7), Qav= 1.3 (n = 50)		
3-spored asci	(28.4) 33.2–39.6 (46.1) × (26.9) 29.6–35.4 (38.2) µm, Av = 36.2 × 32.4µm; Q = 1–1.2 (1.3), Qav = 1.1, (n= 104)	(15.9) 21.3–27.9 (29.9) × (14) 15.9–20.2 (21.9) µm, Av = 33.6 × 24.4 µm; Q = (1) 1.2–1.5 (1.7), Qav = 1.4, (n = 54)		
4-spored asci	(25.6) 28.4–35.9 (38.1) × (22) 23.1–31.3 (34.6) µm, Av = 32.1 × 28.1 µm; Q = (1) 1.1–1.2 (1.4) ; Qav = 1.1, (n = 53)	(16.4) 20.6–26.4 (29.5) × (13.4) 15.9–19.5 (22.4) µm, Av = 23.4 × 17.9 µm; Q = (1) 1.2–1.4 (1.5), Qav = 1.3, (n = 90)		

Shape	subglobose to broadly ellipsoid	broadly ellipsoid to ovoid, often with somewhat pointed apex, shallowly crested to subreticulate	almost exclusively globose to subglobose, however some subglobose ascospores seem slightly tapered/narrowed at the edges, with less than 1% of ascospores fusiform to eye-shaped	subglobose to broadly ellipsoid
Color	cinnamon brown at maturity	at first hyaline, yellowish brown at maturity	orange brown at maturity	orange brown at maturity
Ornamentation	reticulate-alveolate, with broad meshes	with densely arranged, separate, medium sized spines	reticulate-alveolate, with broad meshes	reticulate-alveolate, with broad meshes
Ornamentation height	(3.9) 4.1–5.6 (6.6) µm, Av=4.9 µm (n=30)	(1.3) 1.8–2.6 (3.1) µm, Av=2.2 (n= 46)	(2) 3.1–4.6 (5.3) µm, Av=3.9 µm (n= 81)	(2.5) 3.6–6.3 (7.7) µm, Av=4.9 µm (n= 56)
Meshes shape	regular, closed, irregularly to regularly polygonal (5–6 sides)		regular, closed, irregularly to regularly polygonal with (5–) 6 sides	regular, closed, irregularly to regularly polygonal (5–6 sides)
Meshes lengthwise	4–7 (4 = 18%, 5 = 36%, 6 = 30%, 7 = 16%) (n=50)		3–5 (6) with ratios, 3 = 12%, 4 = 44%, 5 = 38%, 6 = 6% (n=61)	4–7 (8) with ratios 4 = 12%, 5 = 38%, 6 = 34%, 7 = 14%, 8=2% (n=50)
Habitat	in early autumn, under *Pinus sylvestris*	in mid-November, at limestone and bauxite soils, in *A. cephalonica* forest mixed with some scattered *Q. coccifera* shrubs	in spring, under *Tuberaria guttata*	in late autumn to spring, in mountainous areas all over continental Greece, mainly in deciduous forests (*Q. ilex*, *Q. pubescens*, *Corylus*, and/or *Carpinus*), but sometimes also in mixed stands with pines (e.g. *P. halepensis*)

Table S2 (continued)

	T. monosporum (n=1)	***T. regianum*** (n=1)	***T. zambonelliae*** (n=3)
Ascomata			
Diameter	10 x 20 mm	10 mm	12–27 mm
Shape	irregularly globose, tuberiform	globose	globose to lobed
Surface	smooth and shiny	rough, verrucose with small, flat, polygonal or irregularly shaped warts and sparse ridges	smooth
Color	grayish black when semi dried to violaceous black [38]	reddish or blackish-brownish, dark brick [20]	initially buff [52] to ochre-buff, and finally sienna [11] to rusty tawny [14]
Peridium			
Width (n=number of measurements)		(97.6) 123.2–192.7 (226.3) µm, Av: 157.9 µm, (n=41)	(75.5) 109.1–163.1 (191.7) µm thick in total, Av= 136.3 µm, (n=57)

Texture	composed of a densely arranged plectencyma with an external suprapellis with somewhat upraised cystidioid and thick-walled hyphal tips	pseudoparenchymatic, with polygonal large sized cells, composed of an external layer of highly pigmented cells, and an internal layer with hyaline cells	composed an internal layer of hyaline, agglutinated, interwoven hyphae, and an external layer with an intermediate state between very densely agglutinated plectenchymatous texture to pseudoparenchymatic small and irregularly globose cells
External layer dimensions	(27.7) 37.4–60.8 (102.2) µm, Av 49.5 (n=47)	(20.4) 29–58.4 (73.1) µm, Av 41.5 (n=58)	(6.8) 12.1–29.2 (34.3) µm, Av 41.5 (n=58)
External layer texture	plectencymatous dense cutis forming a loosely arranged plectenchyma with somewhat upraised cystidioid and thick-walled hyphal tips	arranged likewards general texture	externally of the outer layer, agglutinated hyaline hyphae, with or without scattered dermatocystidia, especially along the furrows
External layer color	hyaline or faintly pigmented orange brownish	highly pigmented brick brown	hyaline or pigmented brick-brown
Cell dimensions (of pseudoparenchymatic external layer)		(9) 11.1–20.7 (26.6) × (7.2) 9–15.7 (17.5) µm, Av = 15.9 × 12 µm, (n = 100)	(3.7) 4.9–9.2 (11.9) × (3.4) 3.7–7.8 (8.7) µm, Av = 7.2 × 5.8 µm (n = 27)
External elements	cystidioid hyphae (3) 3.5–5 (5.5) µm diameter, straight, septate, ramified, thick walled, membranaceously subyellowish, with cell wall width sometimes up to 1µm		cystidia 14.1–21.16 (21.2) × 4.9–7 µm (n=5)
Gleba			
Texture	firm, solid	firm, solid	firm, solid
Color	same colored with the truffle surface when is semi dryed, grayish black to violaceous black [38]	whitish at first, becoming buff [52] to cinnamon [10], and finally chestnut (23] to dark brick [20] at maturity	whitish at first, towards to cinnamon [10] fawn [29] at maturity
Odor	not recorded	not recorded	not recorded
Asci			
Dimensions (excluding stalk)	(75.9) 87.3–118.3 (144.8) × (61.7) 70.6–96.3 (118.7) µm, Av = 102.9 × 85.1 µm, (n=30)	(48.7) 61.7–71 (74.5) × (47.6) 52.4–61.3 (65.2) µm, Av = 66.8 × 56.8 µm, (n = 40)	(45.9) 52.1–69.2 (82.6) × (29.1) 34.4–49.8 (55.4) µm, Av = 60.3 × 43 µm (n= 44)
Shape	generally subglobose or broadly ellipsoid, without a stalk	irregularly globose, without stalk	mainly pyriform with a long stalk, ranging from irregularly subglobose to nearly fusiform

Stalk dimensions			(26.7) 27.7–46.1 (46.5) × (6.4) 6.6–10.2 (10.3) µm (n=8)
Rate of ascospores per asci	always 1-spored	2 to 8 ascospores per asci, with ratios 2-spored 4%, 3-spored 10%, 4-spored 10%, 5-spored 10%, 6-spored 10%, 7-spored 18%, 8-spored 38% (n=107)	1 to 4 ascospores per asci, with ratios 1-spored 13%, 2-spored 33%, 3-spored 28%, 4-spored 26% (n=100)
Ascospores			
Total dimensions (excluding ornamentation)	(61.6) 67.9–77.3 (81.9) × (53.4) 61.7–72.2 (74.8) µm, Av = 72.7 × 66.8 µm, Q = 1–1.1 (1.2), Qav = 1.1, (n= 79)	of equal size and dimensions regardless of the number of ascospores per ascus (14.0) 16.6–19.5 (21.4) × (11.7) 13.2–15.6 (17.0) µm, Av = 18.5 × 14.25 µm; Q = (1) 1.15–1.41 (1.57), Qav = 1.28, (n = 158)	(20.5) 22.5–29.9 (37.6) × (16.2) 16.8–23.1 (28.2) µm, Av= 26.11 x 19.79 µm, Q = (1) 1.16–1.48 (1.59) Qav=1.32 (n=125)
Total spore volume (only for Regianum Clade truffles–0.523 x W2 x L–excluding ornamentation, according to Merenyi et al. 2017)		(1000) 1572–2318 (3056) µm^3, Vav= 1942 µm^3	
1-spored asci			
2-spored asci			
3-spored asci			
4-spored asci			
Shape	shpaerical, almost exclusively globose to subglobose	broadly ellipsoid	broadly ellipsoid to ellipsoid
Color	dark brick brown	ochre yellowish at maturity	brick brown at maturity
Ornamentation	with hollowed polygonal craters, like inward meshes, resembling a golf ball	reticulate–alveolate, with prominent meshes	densely arranged, separate, medium to large sized spines
Ornamentation height		(1.8) 2–3 (3.6) µm, Av=2.6 µm (n=31)	(2.1) 2.4–3.7 (4.4) µm long, Av= 3µm (n=83)
Meshes shape	Inward meshes irregularly polygonal	regular, closed, polygonal (5–6 sides)	
Meshes lengthwise	(4) 5–8 (9) (n=13)	4–5 (n=41)	

Habitat	in autumn, under *F. sylvatica*	in early autumn, under *F. sylvatica*	in Mediterranean forests or stands dominated by oak species, in mountainous areas, either in the south more xerophilic (e.g. Attica) or in the north more humid habitats; ascomata were found from early winter in northern areas to mid-spring in southern areas.
